# Supplementary material for: Coordination environments of Pt single-atom catalysts from NMR signatures
Source: Nature. 2025 Jun 4;642(8068):613–9. doi: 10.1038/s41586-025-09068-x (PMC12176637; doi:10.1038/s41586-025-09068-x)
Supplement: Supplementary file 1 — Supplementary Sections 1–6, including Supplementary Tables 1–14, Figs. 1–15 and References. [file 41586_2025_9068_MOESM1_ESM.pdf]

nature portfolio

https://doi.org/10.1038/s41586-025-09068-x

Supplementary information

Coordination environments of Pt single-atom catalysts from NMR signatures

In the format provided by the authors and unedited

Supporting Information

Coordination environments of platinum single atom catalysts from NMR signatures

Jonas Koppe[1], Alexander V. Yakimov[2,4], Domenico Gioffrè[2], Marc-Eduard Usteri[2,4], Thomas Vosegaard[3], Guido Pintacuda[1], Anne Lesage[1], Andrew J. Pell[1], Sharon Mitchell[2,4], Javier Pérez-Ramírez[2,4,*], Christophe Copéret[2,4,*]

[1]Centre de RMN Très Hauts Champs de Lyon (UMR5082 - CNRS, ENS Lyon, UCB Lyon 1), Université de Lyon, 5 rue de la Doua, Villeurbanne FR-69100, France

[2]Department of Chemistry and Applied Biosciences, ETH Zürich, Vladimir-Prelog-Weg 1, Zürich CH-8093, Switzerland

[3]Department of Chemistry and Interdisciplinary Nanoscience Center, Aarhus University, Gustav Wieds Vej 14, DK-8000 Aarhus C, Denmark

[4]NCCR Catalysis, Switzerland

E-mail: jpr@chem.ethz.ch; ccoperet@ethz.ch

Table of Contents

Description	Page
1. Conventional characterization of supported samples	S2
2. Experimental ^{195}Pt NMR parameters and data processing	S6
3. Additional ^{195}Pt NMR data	S10
4. DFT computations	S12
5. NMR lineshape model	S15
6. References	S27

1. Conventional characterization of supported samples

1.1 Elemental analysis (EA)

Mikroanalytisches Labor Pascher (An der Pulvermühle 1, 53424 Remagen - Bandorf, Germany) performed the Pt EA via Inductively coupled plasma atomic emission spectroscopy (ICP-AES). EA of other elements (H, C, N, Cl) were measured by the in-house EA service at ETHZ via IR spectrometry (H, C, N; LECO) or Schöniger digestion/Ion Chromatography (Cl).

1.2 N_2 physisorption

N_2 isotherms of the pristine support were measured to derive the total pore (V_{total}), micropore (V_{micro}), and mesopore (V_{meso}) volumes and the total (S_{BET}) and external (S_{ext}) surface areas. The values obtained are typical for nitrogen-doped carbon supports prepared via similar synthetic protocols, ensuring sufficient surface area to stabilize the large number of Pt atoms.

Table S1. Summary of the structural parameters obtained by N_2 adsorption for the pristine carrier.

Sample	V_{total} / cm^3 g^{-1*}	V_{micro} / cm^3 g$^{-1§}$	V_{meso} / cm^3 g$^{-1\#}$	S_{BET} / m^2 g$^{-1ø}$	S_{ext} / m^2 g$^{-1§}$
NC	0.88	0.66	0.22	1474	196

*Determined from the volume of N_2 adsorbed at p/p_0 = 0.99. §Determined by the *t*-plot method. $^\#$Calculated as $V_{meso} = V_{pore} - V_{micro}$. ^{ø}Determined by the BET method.

1.3 X-ray photoelectron spectroscopy

N 1s, Pt 4f, O 1s, and C 1s XPS spectra (**Extended Data Fig. 5**) were measured and fitted (**Table S2** and **Table S3**) for the Pt@NC materials at different loadings (5 and 15 wt%) and synthesis stages (1st and 2nd annealing step), in following referred to as Pt@NC-5-1, Pt@NC-5-2, Pt@NC-15-1, and Pt@NC-15-2, as well as the pure carrier prior to Pt introduction. Pt@NC-15-2$^\#$ refers to the second batch of this sample. Five N moieties were considered for the XPS fitting of the N1s spectra based on previous reports:[1,2] pyridinic N (N_1), pyrrolic N (N_2), oxidized N (N_4), metal-bound N and aminic N. The last two were grouped into a single contribution N_3 to prevent overfitting at the cost of uniqueness of the lineshapes due to insufficient chemical shift separation as recommended by XPS researchers.[1] The fitting shows an almost constant contribution of both N_2 and N_4, whereas an increase in N_3 at the cost of N_2 upon metal introduction and an increase in the loading can be observed (**Table S2**). Accordingly, it may be inferred that Pt atoms preferably bind to pyridinic N moieties as conversion of pyridine N to aminic N seems unlikely. XPS spectra of the other elements shows no changes upon metal introduction and increase of the loading. To determine the Pt oxidation state, all nitrogen and chlorine-containing molecular compounds available on the NIST database[3] were grouped into Pt(II) and Pt(IV), and their binding energies were averaged. This approach was chosen as the binding energies amongst the different Pt(II) and Pt(IV) references were too close. The resulting averages and standard deviations were used to determine the constraints of the fitting. This analysis shows that the Pt is present in a +II oxidation state, consistent with both XAS and NMR. Further changes could not be quantified. Cl could not be analyzed due to a combination of low loading and scan rates.

Table S2. XPS data of N 1s spectra of the different Pt@NC samples as well as the Pt-free carrier.

Sample	Pyridinic-N*			Pyrrolic-N*			N-M/N-H*			N-O*		
	Position / eV	FWHM§ / eV	Area / %	Position / eV	FWHM / eV	Area / %	Position / eV	FWHM / eV	Area / %	Position / eV	FWHM / eV	Area / %
NC	398.2	1.53	47.09	400.1	2.10	40.46	399.1	1.65	7.21	403.5	2.50	5.24
Pt@NC-5-1	398.1	1.42	41.12	400.1	1.95	38.23	399.2	1.59	15.63	403.3	2.50	5.02
Pt@NC-5-2	398.1	1.39	38.84	400.2	2.22	38.71	399.1	1.64	15.99	403.5	2.50	6.46
Pt@NC-15-1	398.1	1.51	35.41	400.2	2.12	39.52	399.1	1.51	20.06	403.5	2.50	5.01
Pt@NC-15-2	398.1	1.32	34.11	400.3	2.20	36.97	399.1	1.44	24.08	403.5	2.50	4.84
Pt@NC-15-2#	398.2	1.28	30.93	400.4	2.22	37.59	399.1	1.50	26.8	403.5	2.50	4.68

*Assigned based on reference values[1,2] §FWHM: full width at half maximum #batch 2

Table S3. XPS fitting data for the Pt 4f spectra of the different Pt@NC samples.

Sample	Pt(IV)*			Pt(II)*			Pt(0)*		
	Position / eV	FWHM§ / eV	Area / %	Position / eV	FWHM / eV	Area / %	Position / eV	FWHM / eV	Area / %
Pt@NC-5-1	75.2	1.85	0	72.5	1.60	100	71.2	2.50	0
Pt@NC-5-2	75.5	2.50	0	72.7	1.49	100	71.2	2.48	0
Pt@NC-15-1	75.3	2.50	0	72.5	1.67	100	71.0	2.44	0
Pt@NC-15-2	75.1	2.50	0	72.6	1.64	100	71.2	2.48	0
Pt@NC-15-2#	75.4	2.32	0	72.7	1.50	100	71.3	2.48	0

*Assigned based on reference values[1,2] §FWHM: full width at half maximum #batch 2

Table S4. Surface concentrations of all elements determined by XPS.

Sample	C / at%	N / at%	O / at%	Pt / at%	Cl / at%	C / wt%	N / wt%	O / wt%	Pt / wt%	Cl / wt%
NC	74.4	20.6	4.2	-	0.3	69.2	22.3	5.2	-	0.7
Pt@NC-5-1	74.7	20.6	3.5	0.3	0.53	69.4	22.3	4.3	5.0	1.5
Pt@NC-5-2	76.4	20.9	1.9	0.3	0.04	71.0	22.6	2.4	5.1	0.1
Pt@NC-15-1	74.6	20.7	2.1	1.0	1.17	69.3	22.4	2.6	15.6	3.2
Pt@NC-15-2	74.6	21.5	2.3	1.0	0.23	69.3	23.3	2.8	15.7	0.6
Pt@NC-15-2*	78.7	17.7	2.1	0.9	0.2	73.1	19.2	2.6	14.2	0.6

*batch 2

1.4 X-ray absorption spectroscopy

X-Ray absorption spectroscopy (XAS) experiments were performed at BM31 of the Swiss-Norwegian Beamlines (SNBL) located at the European Synchrotron Radiation Facility (ESRF) in Grenoble, France. Pt L_3 edge were collected in transmission mode using a double crystal Si (111) monochromator, a

secondary reference (Pt-foil) was used for energy calibration (11564 eV). Typical beam dimensions used were 0.4 mm (H) x 4 mm (W). The sample was packed into quartz capillaries (1.5 mm W, 0.02 mm wall thickness). Spectra were collected at beam energies ranging from 11.46 to-12.36 keV. The scans (5 for molecular samples, 10 for materials) were averaged to obtain a sufficient quality for structural analysis. Demeter software (0.9.24) from the Ifeffit software package (Version 1.2.11) was used for the XAS data analysis.[4]

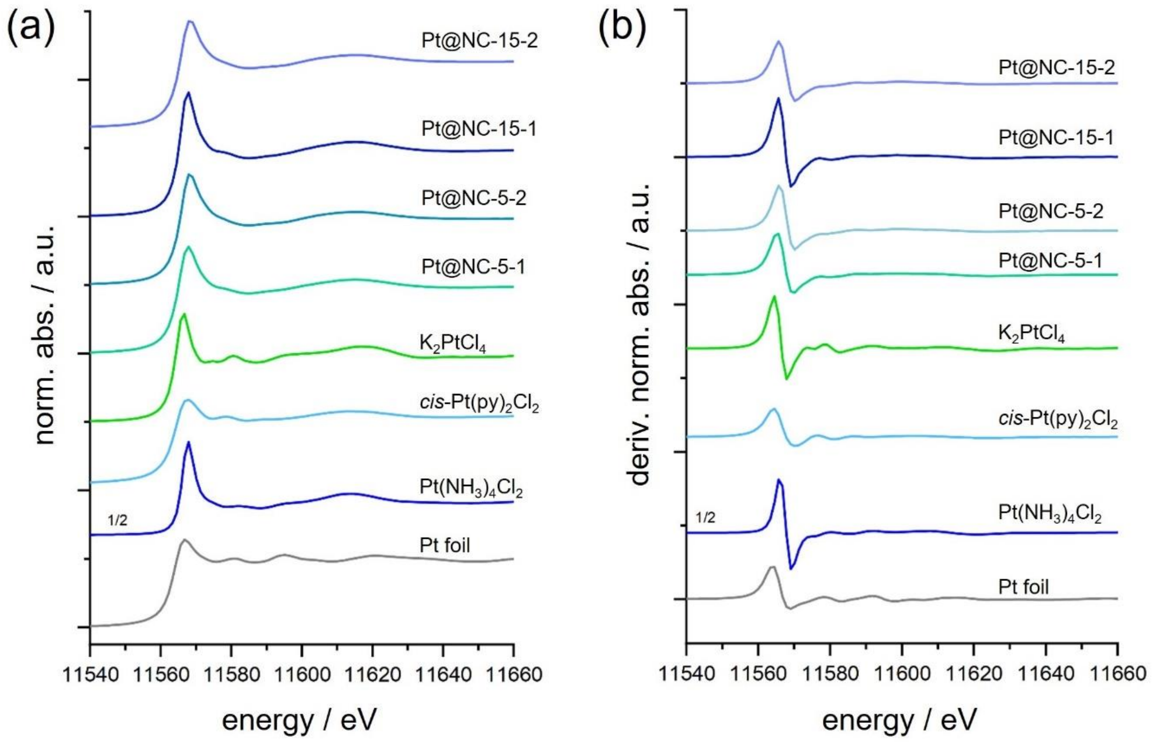

Figure S1. XAS Pt L_3 edge spectra. (a) and their 1st derivative spectra (b) of Pt-foil (metallic), $Pt(NH_3)_4Cl_2$, *cis*-$Pt(py)_2Cl_2$, K_2PtCl_4, and of the Pt@NC materials.

Pt L_3 edge XAS spectra of the Pt-foil (metallic) used as internal reference (11564 eV), a series of molecular complexes with varying Cl content in the 1st Pt coordination environment (K_2PtCl_4, *cis*-$Pt(py)_2Cl_2$, $Pt(NH_3)_4Cl_2$) and the Pt@NC materials with different Pt loadings (5wt%, 15wt%), annealed once and twice, are shown in **Figure S1**. The white line edge energy position (obtained as the highest point of the 1st derivative of the XAS spectra) spans between 11564 and 11566 eV. An increase of 1 eV is observed in the complex with a full-N 1st coordination environment vs. the Cl-containing complexes (comparison between $Pt(NH_3)_4Cl_2$, *cis*-$Pt(py)_2Cl_2$, and K_2PtCl_4). The Pt@NC samples have edge energies between 11565 and 11566 eV, consistent with Pt(II) oxidation state (**Table S5**). The comparison of the couples of materials with the same Pt loading after one or two annealing steps shows an increase in the white line intensity upon a second annealing step, in agreement with the dichlorination of the sample (see EA, **Section S1.1**), consistently to what was observed for the molecular samples. Interestingly, Pt@NC-5 2nd annealing and Pt@NC-15 1st annealing have a similar edge energy, consistent with a comparable Cl/Pt ratio found in the EA (**Section S1.1**).

Table S5: EXAFS Pt L_3 edge energies (eV) at the highest point of each 1st derivative spectrum, as well as distances (Å) at maximum intensity in R-space.

Material	Pt L_3 Edge Energy (eV)	Distance at highest intensity in R-space (Å)
Pt foil (metallic)	11564	2.61
K_2PtCl_4	11564	1.90
cis-Pt(tpy)$_2$Cl$_2$	11564	1.84
$Pt(NH_3)_4Cl_2$	11565	1.62
Pt@NC-5-1	11565	1.66
Pt@NC-5-2	11566	1.56
Pt@NC-15-1	11566	1.66
Pt@NC-15-2	11566	1.53

EXAFS spectra are shown in **Figure S2** for (a) k-space and (b) R-space. EXAFS spectra (k-weight = 3, k-space window ($Å^{-1}$): 3-12 (Pt foil); 3-8 (others)) suggest the absence of metallic Pt (absence of Pt-Pt path, 2.61 Å) for both the molecular samples and the Pt@NC series of materials, in agreement with the presence of isolated Pt sites. A comparison of the materials with the same Pt loadings shows a shift in the distance with highest intensity upon annealing: Pt@NC-5 (1.66 to 1.56 Å) and Pt@NC-15 (1.66 to 1.53 Å). This is consistent with the trend observed among the three molecular complexes, for which maxima in the distance are found to be at 1.90 Å (K_2PtCl_4), 1.84 Å (*cis*-Pt(py)$_2$Cl$_2$), and 1.62 Å ($Pt(NH_3)_4Cl_2$), going from 4 to 0 Cl atoms in the 1st coordination shell of Pt (**Table S5**). The decrease in intensity of the Pt-Cl path (1.90 Å) and an increase in the Pt-N path (1.62 Å) upon a second annealing step is therefore consistent with the de-chlorination of the samples, and changes in the 1st coordination environment of Pt upon annealing.

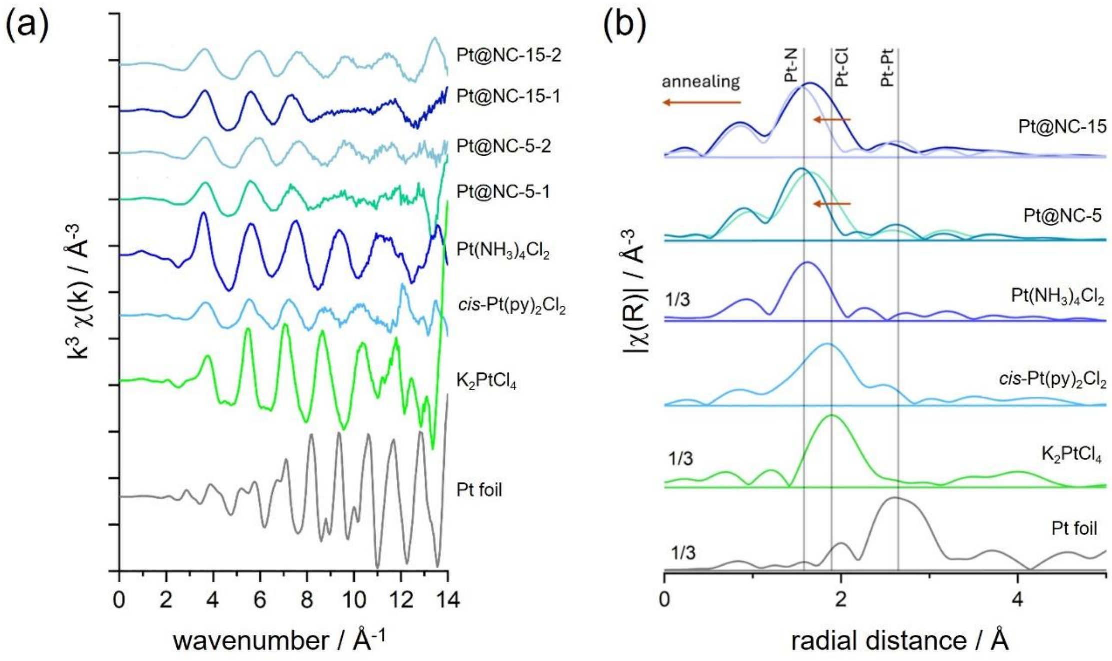

Figure S2. EXAFS Pt L_3 edge spectra. Pt-foil (metallic), $Pt(NH_3)_4Cl_2$, *cis*-Pt(py)$_2$Cl$_2$, K_2PtCl_4, and of the Pt@CN materials: (a) k-space and (b) R-space. In (b), vertical lines indicate distances (Å) of: 2.61 (Pt-Pt); 1.90 (Pt-Cl); 1.62 (Pt-N).

2. Experimental ^{195}Pt NMR parameters and data processing

^{195}Pt NMR spectra have been recorded using the WCPMG pulse sequence under static and MAS conditions shown in **Figure S3**.[5,6]

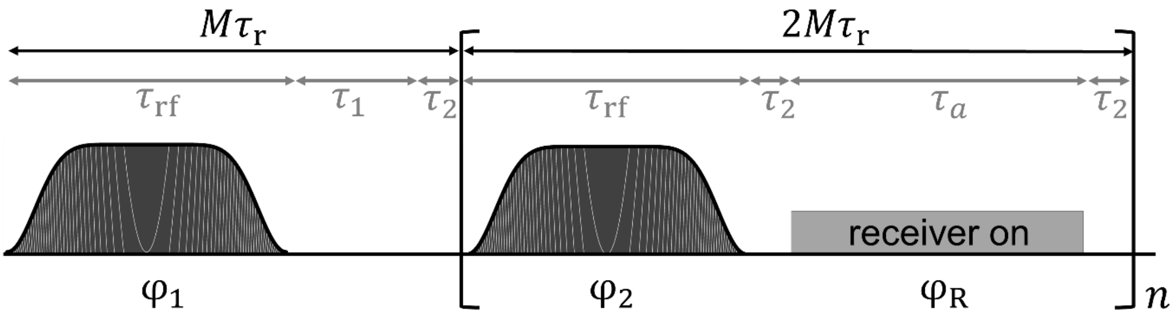

Figure S3. WCPMG NMR pulse sequence for static and MAS conditions. Identical WURST-N pulses of length τ_{rf} are used, and the acquisition time is denoted as τ_a. A short ringdown delay τ_2 can be incorporated to reduce pulse artifacts. The number of CPMG echoes is given by *n*. The pulse phases are set according to $\phi_1 = \phi_2 - \frac{\pi}{2} = \phi_R$, where ϕ_R represents the receiver phase. The first echo delay is set to $\tau_1 = (\tau_a - \tau_{rf})/2$.[7] Under MAS conditions, the sequence is synchronized with the rotation, where τ_r denotes the rotation period.[6] Then, the first echo delay must be set to $\tau_1 = M\tau_r - \tau_{rf}$, where *M* is an integer number, defining the number of rotor echoes recorded with each echo in the CPMG train. Rotor synchronization is ensured by setting the acquisition time to $\tau_a = 2M\tau_r - \tau_{rf} - 2\tau_2$. A minimum phase cycle should be employed for optimum S/N.[7]

The ^{195}Pt NMR measurements (static and 20 kHz MAS) on the molecular compounds K_2PtCl_4, *cis*-$Ptpy_2Cl_2$, $Pt(NH_3)_4Cl_2$, and *cis*-$PtMe_2$tmeda were conducted at room temperature in a single offset using *continuous-wave* ^{1}H decoupling (20 kHz). For K_2PtCl_4, two offsets were required (spaced by 250 kHz), and no ^{1}H decoupling was applied. The experimental parameters are again summarized in **Table S6** and **Table S7**.
The ^{195}Pt NMR measurements (static and 10 kHz MAS) on the SACs were conducted at low temperature (100 K) using two offset (spaced by 250 kHz). The spectrum of Pt@PTI was recorded at a single offset only. The experimental parameters are again summarized in **Table S8**, **Table S9**, and **Table S10**.
All ^{195}Pt NMR spectra were obtained by coadding the individual echoes of the WCPMG-echo train in the time domain prior to Fourier transform and magnitude calculation. Typically, the first, or the first two echoes were omitted due to ring-down voltages. The resulting full echoes were apodised using a Hann or Hamming window function[8] with α = 0.54 (see **Figure S4**). This is illustrated in **Figure S5**.

When the full ^{195}Pt NMR signatures were reconstructed from two subspectra, the skyline projection method was used. For the sample Pt@NC-5 (2nd annealing), the second subspectrum (static and MAS) needed to be recorded with a freshly packed rotor, due to a rotor crash at the end of the acquisition of the first subspectrum. To compensate for the difference in sample volume, the intensities for both subspectra were scaled to match at the center in between their frequency offsets (cf. **Figure S7**). This resulted in a down-scaling of the second subspectrum (static and MAS) by 10 %.
We note that in the ^{195}Pt MAS NMR signals of the Pt@NC samples, no rotor echoes were observed due to the Pt-site heterogeneity, and ultimately large distribution of ^{195}Pt isotropic chemical shifts. Therefore, the full echo (after coadding all individual echoes from WCPMG train) was drastically apodised to reduce the noise. The full procedure is demonstrated in **Figure S5**.

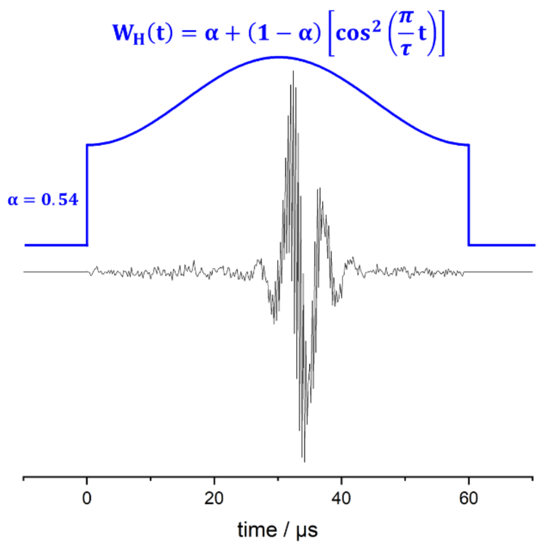

Figure S4. Illustration of the Hamming window function WH(t) used to process the coadded full echoes obtained from the WCPMG train. Here, τ is the window length and is set to match the full echo length (60 µs in this example). The truncation parameter α was set to α = 0.54, offering a good compromise between the obtained linewidth and the signal-to-noise.[8]

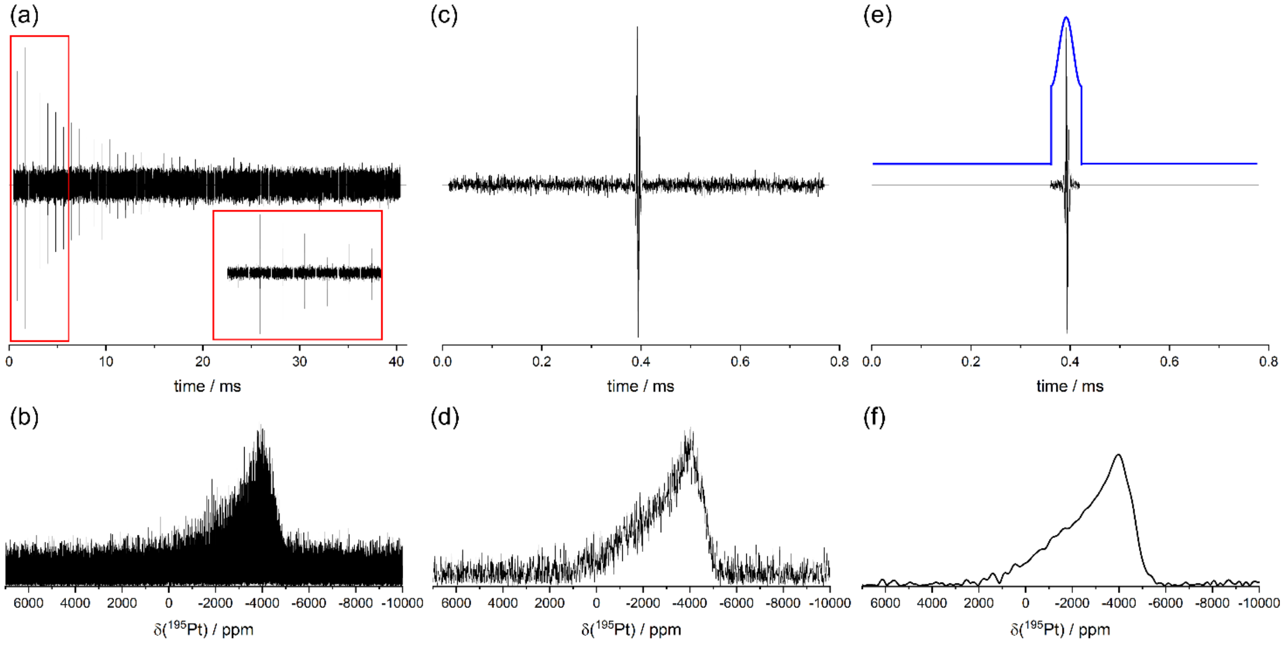

Figure S5. Processing procedure for WCPMG-MAS spectra. (a) Raw time-domain signal, i.e., the full WCPMG train with 50 echoes of length 730 µs, theoretically comprising 7 rotor echoes each (three rotor echoes for the built-up part, the central echo, and three rotor echoes in the decaying part). (b) shows the corresponding frequency-domain spectrum obtained after FT of the full FID. (c) The full echo obtained from coadding the first 30 echoes from the WCPMG train in (a). Clearly, no rotor echoes are observed, such that most of the echo contains only noise, as can also be seen from (d), the corresponding spectrum in the bottom panel. (e) Echo from (c), apodised to match the echo length used in the static WCPMG experiments (60 µs), combined with the Hamming window function in blue. (f) shows the final ^{195}Pt WCPMG-MAS spectrum as reported in the main text. The exemplified data here correspond to the first offset for high Pt-loading samples Pt@NC (batch 2, **Figure 4a** in the main text).

Table S6. Static ^{195}Pt WCPMG NMR experiments on the molecular compounds in **Figure 2a-d**. The four numbers in a single line refer to the individual compounds in the following order: K_2PtCl_4, cis-$Ptpy_2Cl_2$, $Pt(NH_3)_4Cl_2$, and cis-$PtMe_2$tmeda.

Larmor frequency (MHz)	86.1183
Recycle delay (s)	30, 30, 4, 30
Dwell time (μs)	0.2
Processed echoes	200, 100, 75, 10
Number of scans per offset$^{\&}$	1880, 5200, 4096, 2240
Number of offsets$^{\$}$	1
Echo delay τ_1 (μs)	60
Ring-down delay τ_2 (μs)	10
WURST-pulse length (μs)	30
Sweep width (kHz)	1400
WURST-pulse shape parameter, N	10
Nutation frequency$^{\#}$ (kHz)	57

$^{\&}$Some experiments have larger number of scans than required due to overnight runtimes

$^{\$}$Two offsets were recorded for K_2PtCl_4.

$^{\#}$The ^{195}Pt nutation frequency was calibrated using K_2PtCl_6.

Table S7. ^{195}Pt WCPMG-MAS NMR (20 kHz) experiments on the molecular compounds in **Figure 2a-d**. The four numbers in a single line refer to the individual compounds in the following order: K_2PtCl_4, cis-$Ptpy_2Cl_2$, $Pt(NH_3)_4Cl_2$, and cis-$PtMe_2$tmeda.

Larmor frequency (MHz)	86.1183
Recycle delay (s)	30, 30, 4, 30
Dwell time (μs)	0.2
Processed echoes	100, 100, 75, 10
Number of rotor echoes	5
Number of scans per offset$^{\&}$	48, 1888, 15360, 216
Number of offsets$^{\$}$	1
Echo delay τ_1 (μs)	230
Ring-down delay τ_2 (μs)	10
WURST-pulse length (μs)	50
Sweep width (kHz)	3000
WURST-pulse shape parameter, N	2
Nutation frequency$^{\#}$ (kHz)	65

$^{\&}$Some experiments have larger number of scans than required due to overnight runtimes

$^{\$}$Two offsets were recorded for K_2PtCl_4.

$^{\#}$The ^{195}Pt nutation frequency was calibrated using K_2PtCl_6.

Table S8. Static ^{195}Pt WCPMG NMR experiments on the SACs in **Figure 2e-h**., and **Figure 4a** and **b.** The six numbers in a single line refer to the individual samples in the following order: Pt@NC-5 first and second annealing, Pt@NC-15 first and second annealing, Pt@NC (batch 2), Pt@PTI, and Pt@SiO_2.

Larmor frequency (MHz)	86.1183
Recycle delay (s)	0.2
Dwell time (μs)	0.2
Processed echoes	100
Number of scans per offset$^{\&}$	1303632, 225536, 47672, 37520, 368967, 404744, 436272
Number of offsets$^{\$}$	2
Echo delay τ_1 (μs)	60
Ring-down delay τ_2 (μs)	10
WURST-pulse length (μs)	30
Sweep width (kHz)	1400
WURST-pulse shape parameter, N	10
Nutation frequency$^{\#}$ (kHz)	57

$^{\&}$Some experiments have larger number of scans than required due to overnight runtimes

$^{\$}$One offset was recorded for Pt@PTI.

$^{\#}$The ^{195}Pt nutation frequency was calibrated using K_2PtCl_6.

Table S9. ^{195}Pt WCPMG-MAS NMR (10 kHz) experiments on the SACs in **Figure 2e-h**. The four numbers in a single line refer to the individual samples in the following order: Pt@NC-5 first and second annealing, Pt@NC-15 first and second annealing.

Larmor frequency (MHz)	86.1183
Recycle delay (s)	0.2
Dwell time (μs)	0.2
Processed echoes	20, 40, 50, 50
Number of rotor echoes	3
Number of scans per offset$^{\&}$	833696, 385496, 66952, 65536
Number of offsets	2
Echo delay τ_1 (μs)	230
Ring-down delay τ_2 (μs)	10
WURST-pulse length (μs)	50
Sweep width (kHz)	3000
WURST-pulse shape parameter, N	2
Nutation frequency$^{\#}$ (kHz)	65

$^{\&}$Some experiments have larger number of scans than required due to overnight runtimes

$^{\#}$The ^{195}Pt nutation frequency was calibrated using K_2PtCl_6.

Table S10. Static ^{195}Pt WCPMG NMR experiments on the SACs in **Figure 4c.** The three numbers in a single line refer to the individual samples in the following order: Pt@NC-1 second annealing pristine material, after 1 h on stream, after 12 h on stream.

Larmor frequency (MHz)	86.1183
Recycle delay (s)	0.2
Dwell time (μs)	0.2
Processed echoes	200, 100, 50
Number of scans per offset	160960, 281232, 668256
Number of offsets	2
Echo delay τ_1 (μs)	60
Ring-down delay τ_2 (μs)	10
WURST-pulse length (μs)	30
Sweep width (kHz)	1400
WURST-pulse shape parameter, N	10
Nutation frequency$^{\#}$ (kHz)	57

$^{\#}$The ^{195}Pt nutation frequency was calibrated using K_2PtCl_6.

3. Additional ^{195}Pt NMR data

Notably, direct detection via ^{195}Pt WCPMG NMR of single Pt atoms without any further, more advanced sensitivity-enhancement protocols (e.g., dynamic nuclear polarization) is enabled by the combined effects of low experimental temperatures (100 K) and very fast repetition rates (0.2 s). While this combination has already been exploited by Slichter and co-workers in their pioneer work on *metallic* Pt,[9–12] such short longitudinal relaxation (T_1) times are atypical for Pt(II). The longitudinal relaxation behavior and the effect of the temperature on the ^{195}Pt NMR lineshape is demonstrated for the example of the high Pt-loading sample Pt@NC (batch 2; from **Figure 4a** of the main text) in **Extended Data Fig. 1**. Neither the short repetition rates (see **Extended Data Fig. 1**a-c) nor the low temperatures (see **Extended Data Fig. 1**d) do impact significantly on the lineshape. Generally, low temperatures may have a deleterious effect on the ^{195}Pt T_1 relaxation times of molecular Pt(II) compounds, which in many cases even at room temperature exhibit unfavorable T_1 relaxation times (30 s and more). However, we suspect that due to the (partial) integration of the Pt centers into the surface of the support material, the surface dynamics, modulating the large shift anisotropies, cause efficient ^{195}Pt spin-lattice relaxation even at low temperatures. This is further corroborated by the fact that the Pt@NC sample with the (presumably) least degree of Pt surface integration (Pt@NC-5, first annealing) required the longest experimental times (1.5 days per subspectrum), while samples with equivalent Pt-loading (Pt@NC-5, second annealing) or even less (Pt@PTI, 2 wt%, or Pt@NC-1 after the second annealing) but a (presumably) better Pt surface integration could be recorded in significantly shorter times (less than one day).

We note that since the nitrogen-doped carbon support is conductive, a hyperfine coupling between the conduction electrons of the support and the ^{195}Pt nuclei would potentially induce a ^{195}Pt Knight shift K.[13] This Knight shift would sum to the chemical shift and therefore complicate the data analysis. This same hyperfine interaction would also give a contribution to the spin-lattice relaxation rate R_1, which is related to the Knight shift via the Korringa relation,[14] i.e., $K^2 \sim R_1$. Therefore, if the Knight shift does represent a significant contribution to the ^{195}Pt NMR lineshape, we would observe a correlated distribution of K and R_1 across the resonance. In particular, on reducing the recycle delay we would observe differential saturation of the signal intensity versus the shift. However, from **Extended Data Fig. 1**b and c, it is clear that the saturation we observe is uniform across the lineshape, and so there is no significant Korringa behavior, which further implies that the contribution from the Knight shift to the chemical shift is negligible, if present at all.

We also tested the effect of CPMG acquisition on the ^{195}Pt NMR spectra. Again, we found that the lineshape integrity is fully maintained, as demonstrated in **Figure S6**. As it was discussed in the main text, the Pt-site heterogeneity causes a Gaussian distribution of ^{195}Pt isotropic chemical shifts much larger than the applied MAS frequency (10 kHz). Therefore, we evaluated the effect of fast spinning (50 kHz MAS) on the lineshape for the high Pt-loading sample Pt@NC (batch 2, as already shown above). The ^{195}Pt WCPMG MAS spectrum (50 kHz MAS; first subspectrum) is shown in **Extended Data Fig. 6**. Based on the Monte-Carlo simulation of the static lineshape shown in **Figure 4a** in the main text, we expect a standard deviation of ^{195}Pt isotropic chemical shift of 760 ppm, at the order of the MAS frequency (50 kHz ≈ 600 ppm). The spectrum indeed appears to show slight narrowing, and the experimental and numerical data are in close agreement, further supporting the lineshape model developed in this study.

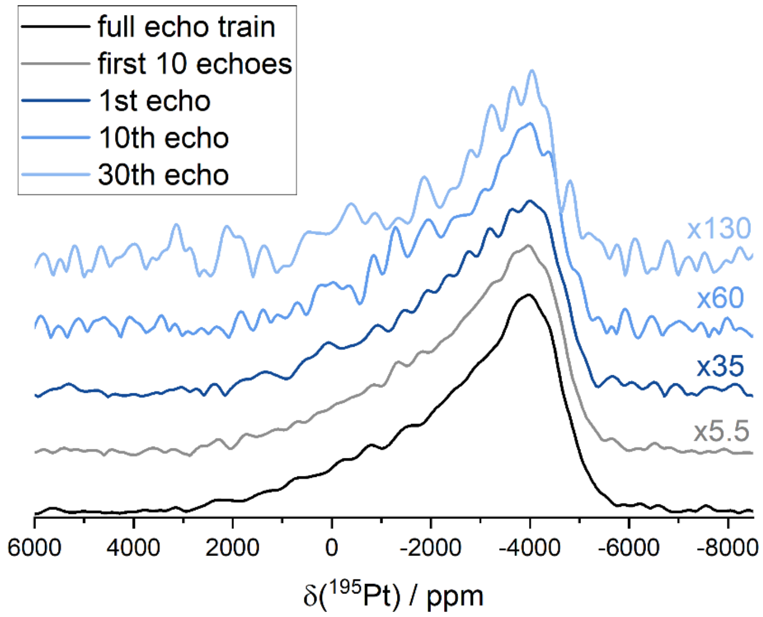

Figure S6. Effect of the CPMG acquisition on the static ^{195}Pt NMR signature of Pt@NC (batch 2). A comparison of the spectra resulting from FT and magnitude calculation of the echo obtained from adding up the first 100 WCPMG echoes (full echo train), the first 10 WCPMG echoes, as well as from the first, the 10th, and the 30th WCPMG echo only.

Lastly, we show the effect of including a second offset (a second subspectrum) in the re-construction of the overall ^{195}Pt NMR signatures of the samples containing single Pt atoms. This is demonstrated for the sample Pt@NC-5 (first annealing; **Figure 2**e in the main text), showing the largest extension towards higher frequencies (towards the left) of all samples. As a reference, the frequency axis (in MHz) is likewise indicated. The carriers (subspectrum centers) are demonstrated by the arrows.

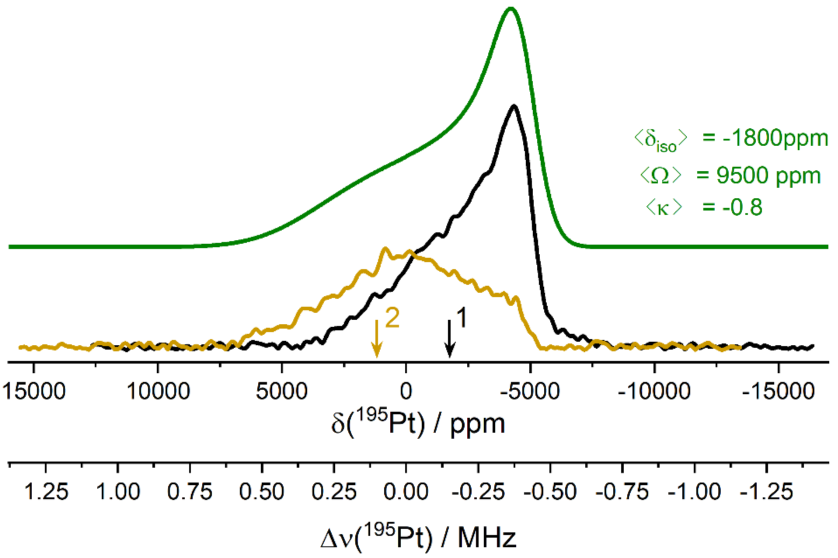

Figure S7. Effect of including a second subspectrum in the re-construction of the overall static ^{195}Pt NMR signature of Pt@NC-5 (first annealing).

4. DFT computations

Geometry optimization of a series of Pt(II) model complexes and calculations of their ^{195}Pt NMR spectroscopic parameters were performed using ADF 2022 with the hybrid PBE0 functional and Slater-type basis sets of quadruple-ζ quality (QZ4P) for Pt, triple-ζ quality (TZP) for the Pt first coordination shell and double-ζ quality (DZP) for other atoms[15]. Relativistic effects were treated by the two-component zeroth order regular approximation (ZORA)[16–18].

4.1 Benchmarking

^{195}Pt NMR computations have been benchmarked using a library of seven molecular Pt(II) square-planar complexes with δ_{iso} ranging between -360 and -3840 ppm and Ω going from 4600 up to 12521 ppm (**Table S11**). The calculated shielding values of the benchmark structures yielded good agreement (linear relationship) with the experimental chemical shift values (**Figure S8**); a good agreement between the experimental and DFT Ω values is also found (**Figure S9**), coherent with previous reports.[19,20] The cartesian coordinates of the optimized structures are found as an *addendum*.

Table S11*:* Molecular complexes used to benchmark DFT calculations. Experimental NMR parameters were obtained in this work (*a*), or reported in our previous collaborative work[19] (*b*) and by Schurko and collaborators[21] (*c*).

Molecule	σ_{iso} (DFT)	δ_{iso} (experiment)	Ω (DFT)	Ω (experiment)
$Pt(acac)_2$	2384	-360	12200	12521[b]
K_2PtCl_4	3194	-1650[a]	10586	9080[a]
cis-$Pt(NH_3)_2Cl_2$	4307	-1825	7473	8975[c]
cis-$Pt(py)_2Cl_2$	3892	-2065[a]	6085	8500[a]
trans-$Pt(NH_3)_2Cl_2$	4120	-2200	8770	9100[c]
$[Pt(NH_3)_4]Cl_2$	4589	-2540[a]	7078	7220[a]
(tmeda)$PtMe_2$	5827	-3840[a]	4173	4600[a]

4.2 DFT study on the effect of chemical environment on ^{195}Pt NMR parameters

We carried out a systematic study on the response of the ^{195}Pt NMR parameters (δ_{iso}, Ω) of a series of Pt(II) square-planar model complexes towards the Pt chemical environment (**Extended Data Fig. 7**, **Table S12**). The cartesian coordinates of the optimized structures are found as an *addendum*. δ_{iso} values were obtained referencing the computed σ_{iso} values *via* the obtained linear relationship:

$$\delta_{iso} = -0.93 * \sigma_{iso} + 1690\ ppm$$ (see **Section S4.1** for details)

Specifically, we considered a variety of ligands, coordinating the metal *via* O, C, N and C. We evaluated the effect of the different N and C ligands, including both aromatic (pyridine, pyrazine, benzene) and non-aromatic (ammonia, methyl) ones. The effects of chelating ligands (bipyridine, bipyrazine, biphenyl, tmeda) as well as that of possible isomers (*cis vs. trans*) were also taken into account. Overall, we found that the ^{195}Pt NMR parameters are related to specific coordination environments. The highest δ_{iso}/Ω are associated to a O_4 environment (-525/12200), both values decrease for Cl_4 environment (- 1278/9080). Further lower δ_{iso}/Ω values are observed in the series of N_2Cl_2 model compounds studied; these species cluster in the light-blue area of the δ_{iso}/Ω map (-1914 > δ_{iso} > -2353); 8770 > Ω > 4254), showing that the nature of the N-based ligands and of structural isomers can further modulate the NMR parameters. The family of N_4 structures (blue area) showed lower δ_{iso} values; the NMR parameters of these species appear to be only slightly sensitive to changes in the nature of the N-ligands (-2530 ppm > δ_{iso} > -2668 ppm; 7644 ppm > Ω > 6118 ppm). In contrast, the N_2C_2 species (grey area) show a large variability in δ_{iso} values (-2640 > δ_{iso} > -3941), while they all share relatively low Ω values (4278 > Ω > 2576). Similar trends have already been observed in previous studies.[19,20] Computed NMR parameters

for the Pt@SiO_2-C_2O_2 environment were reported in our previous work (δ_{iso}:-2959 ppm; Ω: 6703 ppm; κ:-0.97).[19]

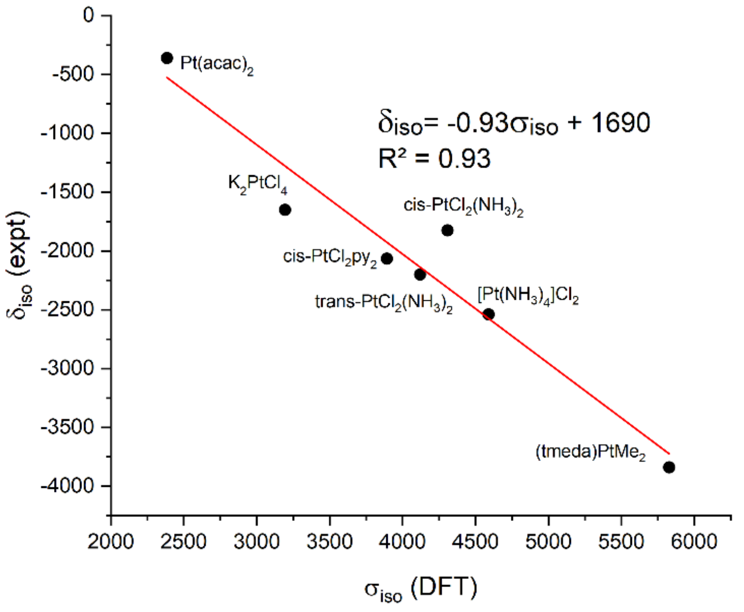

Figure S8. Correlation between experimental chemical shift δ_{iso}(^{195}Pt) and calculated chemical shielding σ_{iso}(^{195}Pt) for a set of seven Pt(II) square-planar complexes. The linear relation is found to be δ_{iso}= -0.93 * σ_{iso} + 1690 ppm (R^2 = 0.93).

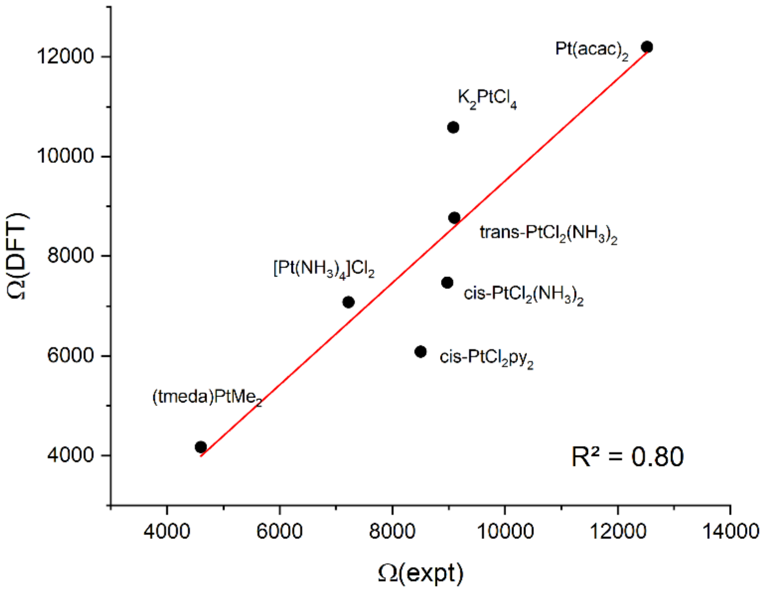

Figure S9. Correlation between experimental and calculated Ω values for a set of seven Pt(II) square-planar complexes.

Table S12: ^{195}Pt NMR parameters calculated for the **1-21** structures shown in **Extended Data Fig. 7**.

Structure	σ_{iso} (DFT)	δ_{iso} (DFT, referenced)	Ω (DFT)
**1**	2384	-525	12200
**2**	3194	-1278	9080
**3**	3878	-1914	6667
**4**	4083	-2104	7835
**5**	4120	-2138	8770
**6**	4308	-2313	7473
**7**	4351	-2353	4254
**8**	4542	-2530	6250
**9**	4543	-2532	7644
**10**	4558	-2546	6118
**11**	4589	-2575	7400
**12**	4659	-2640	4278
**13**	4688	-2667	2737
**14**	4690	-2668	7476
**15**	4850	-2817	4212
**16**	5093	-3043	3898
**17**	5574	-3490	4233
**18**	5589	-3504	3038
**19**	5641	-3552	3215
**20**	5827	-3725	4173
**21**	6059	-3941	2576

5. NMR lineshape model

5.1 General considerations

In the following, we indicate scalar deterministic variables as A, and random variables as A, where the superscript *(j)* is introduced, i.e., $A^{(j)}$, to identify an explicit event for the random variable A. The corresponding tensorial quantities are denoted as in $\mathbf{A}$, $\boldsymbol{A}$, and $\boldsymbol{A}^{(j)}$.
Under high-field conditions, the chemical shift (CS) tensor $\boldsymbol{\delta}$ can be described by a symmetric, second-rank Cartesian tensor within good approximation, and is therefore fully defined by six components,

$$\boldsymbol{\delta} = \begin{bmatrix} \delta_{11} & \delta_{12} & \delta_{13} \\ \delta_{12} & \delta_{22} & \delta_{23} \\ \delta_{13} & \delta_{23} & \delta_{33} \end{bmatrix}. \tag{1}$$

In principle, a random CS tensor $\tilde{\boldsymbol{\delta}}$ can thus be construct from six random variables.[22] For the distribution of j ^{195}Pt sites, the corresponding ^{195}Pt NMR signatures can be described by the sum of individual NMR signals due to all CS tensors $\boldsymbol{\delta}^{(j)}$. For the model employed here, we assume that each $\boldsymbol{\delta}^{(j)}$ comprises two contributions, and can be expressed as

$$\boldsymbol{\delta}^{(j)} = \boldsymbol{\delta}^{\mathbf{0}} + \tilde{\boldsymbol{\delta}}^{(j)} = \begin{bmatrix} \langle\delta_{11}\rangle & 0 & 0 \\ 0 & \langle\delta_{22}\rangle & 0 \\ 0 & 0 & \langle\delta_{33}\rangle \end{bmatrix} + \begin{bmatrix} \Delta\delta_{11}^{(j)} & \Delta\delta_{12}^{(j)} & \Delta\delta_{13}^{(j)} \\ \Delta\delta_{12}^{(j)} & \Delta\delta_{22}^{(j)} & \Delta\delta_{23}^{(j)} \\ \Delta\delta_{13}^{(j)} & \Delta\delta_{23}^{(j)} & \Delta\delta_{33}^{(j)} \end{bmatrix}, \tag{2}$$

implying that that $\boldsymbol{\delta}^{(j)}$ is described in a frame of reference where $\boldsymbol{\delta}^{\mathbf{0}}$ is diagonal. We note the close resemblance to the extended Czjzek model[22,23] (see discussion below), where $\boldsymbol{\delta}^{\mathbf{0}}$ is referred to as the local contribution that is identical for all sites, and $\tilde{\boldsymbol{\delta}}^{(j)}$ (apart from the scaling factor, see Eq. (10)[22,23]) was termed the noise or background contribution, that varies across the different local environments. Within the model introduced in this study, $\boldsymbol{\delta}^{\mathbf{0}}$ contains information about the average or dominant local Pt(II) environment (in the later analysis determining the peak center in δ_{iso}-Ω-space), while $\tilde{\boldsymbol{\delta}}^{(j)}$ comprise information about Pt(II)-site heterogeneity (the peak width and extension in δ_{iso}-Ω-space). To simplify the model in Eq. (2) and to reflect the close to axially symmetric (oblate) of square-planar Pt(II) sites, we approximate $\boldsymbol{\delta}^{(j)}$ by

$$\boldsymbol{\delta}_{sq}^{(j)} = \begin{bmatrix} \langle\delta_{11}\rangle & 0 & 0 \\ 0 & \langle\delta_{22}\rangle & 0 \\ 0 & 0 & \langle\delta_{33}\rangle \end{bmatrix} + \begin{bmatrix} U_{\mathrm{long}}^{(j)} & 0 & 0 \\ 0 & U_{\mathrm{eq}}^{(j)} & 0 \\ 0 & 0 & U_{\mathrm{eq}}^{(j)} \end{bmatrix}, \tag{3}$$

where $\langle\delta_{11}\rangle \geq \langle\delta_{22}\rangle \geq \langle\delta_{33}\rangle$, and $U_{\mathrm{long}}^{(\mathrm{j})}$ and $U_{\mathrm{eq}}^{(j)}$ are treated as independent random variables, described by Gaussian distributions $U_{\mathrm{long}}^{(\mathrm{j})} \sim \mathcal{N}(0, \sigma_{\mathrm{long}}^2)$ and $U_{\mathrm{eq}}^{(j)} \sim \mathcal{N}(0, \sigma_{\mathrm{eq}}^2)$, i.e., with expectation values $\langle U_{\mathrm{long}}^{(\mathrm{j})}\rangle = \langle U_{\mathrm{eq}}^{(j)}\rangle = 0$, and different standard deviations for the longitudinal and equatorial components, σ_{long} and σ_{eq}, respectively.[24] We can combine the two contributions in Eq. (3) and write

$$\boldsymbol{\delta}_{sq}^{(j)} = \begin{bmatrix} \Delta_{11}^{(j)} & 0 & 0 \\ 0 & \Delta_{22}^{(j)} & 0 \\ 0 & 0 & \Delta_{33}^{(j)} \end{bmatrix}, \tag{4}$$

with $\Delta_{11} \sim \mathcal{N}(\langle\delta_{11}\rangle, \sigma_{\mathrm{long}}^2)$, $\Delta_{22} \sim \mathcal{N}(\langle\delta_{22}\rangle, \sigma_{\mathrm{eq}}^2)$, and $\Delta_{33} \sim \mathcal{N}(\langle\delta_{33}\rangle, \sigma_{\mathrm{eq}}^2)$. We note that this approach is very similar as the application of an anisotropic line broadening, as it is for example known from electron paramagnetic resonance to model a distribution of spin Hamiltonian parameters.[25] Generally,

for an oblate CS-tensor shape ($\kappa \approx 1$) as expected for the square-planar Pt(II) structural motif common for all Pt sites, $\langle\delta_{11}\rangle \gg \langle\delta_{22}\rangle \approx \langle\delta_{33}\rangle$.

The individual CS-tensor components, the isotropic shift $\delta_{\mathrm{iso}}^{(j)}$, and span $\Omega^{(j)}$, and the skew $\kappa^{(j)}$, are calculated according to[26]

$$\begin{aligned} \delta_{\mathrm{iso}}^{(j)} &= \frac{1}{3}\left(\Delta'^{(j)}_{11} + \Delta'^{(j)}_{22} + \Delta'^{(j)}_{33}\right) \\ \Omega^{(j)} &= \Delta'^{(j)}_{11} - \Delta'^{(j)}_{33} \\ \kappa^{(j)} &= \frac{3\left(\Delta'^{(j)}_{22} - \delta_{\mathrm{iso}}^{(j)}\right)}{\Omega^{(j)}}, \end{aligned} \tag{5}$$

where the three CS-tensor components from Eq. (4) are re-assigned according to $\Delta'^{(j)}_{11} \geq \Delta'^{(j)}_{22} \geq \Delta'^{(j)}_{33}$ if required, since not necessarily $\Delta^{(j)}_{11} \geq \Delta^{(j)}_{22} \geq \Delta^{(j)}_{33}$ for each $\boldsymbol{\delta}_{sq}^{(j)}$. The marginal distribution of the random variable δ_{iso} is likewise a Gaussian distribution,

$$\delta_{\mathrm{iso}} \sim \mathcal{N}\left(\frac{1}{3}(\langle\delta_{11}\rangle + \langle\delta_{22}\rangle + \langle\delta_{33}\rangle), \frac{1}{9}\left(\sigma_{\mathrm{long}}^2 + 2\sigma_{\mathrm{eq}}^2\right)\right). \tag{6}$$

Analogously, the marginal distribution for the span can also be described by Gaussian distribution,

$$\Omega \sim \mathcal{N}\left(\langle\delta_{11}\rangle - \langle\delta_{33}\rangle, \left(\sigma_{\mathrm{long}}^2 + \sigma_{\mathrm{eq}}^2\right)\right), \tag{7}$$

however only for the case that for all $\boldsymbol{\delta}_{sq}^{(j)}$ the original assignment $\Delta^{(j)}_{11} \geq \Delta^{(j)}_{22} \geq \Delta^{(j)}_{33}$ remains valid. In the case of square-planar Pt(II), Eq. (7) is still approximately true as long as $\Omega \gg \sigma_{\mathrm{long}} > \sigma_{\mathrm{eq}}$.

5.2 Evaluating the distribution of the CS tensors

5.2.1 Representations of the distribution of CS tensors. The distribution of CS tensors $\boldsymbol{\delta}_{sq}^{(j)}$, that results from the five input parameters $\langle\delta_{11}\rangle$, $\langle\delta_{22}\rangle$, $\langle\delta_{33}\rangle$, σ_{long}, and σ_{eq}, can be conveniently evaluated by Monte-Carlo simulations. To this end, 10^7 individual CS tensors $\boldsymbol{\delta}_{sq}^{(j)}$ are constructed, and the three components $\Delta^{(j)}_{11}$, $\Delta^{(j)}_{22}$, and $\Delta^{(j)}_{33}$ are re-ordered into $\Delta'^{(j)}_{11} \geq \Delta'^{(j)}_{22} \geq \Delta'^{(j)}_{33}$. For each $\boldsymbol{\delta}_{sq}^{(j)}$, the three CS-tensor parameters $\delta_{\mathrm{iso}}^{(j)}$, $\Omega^{(j)}$, and $\kappa^{(j)}$ are calculated according to Eq. (5). The distribution of the CS tensors along the dimensions δ_{iso}, Ω, and κ, therefore in the following denoted as $\mathrm{P}(\delta_{\mathrm{iso}}, \Omega, \kappa) \equiv \mathrm{P}(\boldsymbol{\delta}_{sq})$, is obtained by computing a three-dimensional (3D) histogram with voxels (δ_{iso},Ω,$\kappa \equiv \boldsymbol{\delta}_{\mathrm{sq}}$). Then, each voxel ($\boldsymbol{\delta}_{\mathrm{sq}}$) represents a unique ^{195}Pt NMR signal, and its entry indicates the number of appearances (counts) of the associated CS tensor $\boldsymbol{\delta}_{sq}$ among the 10^7 individual $\boldsymbol{\delta}_{sq}^{(j)}$.

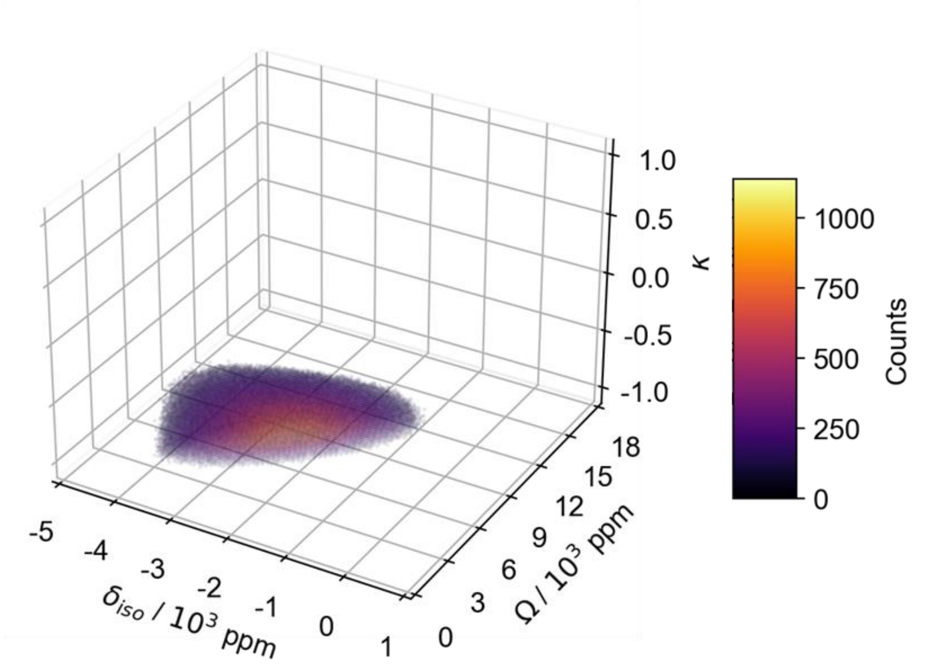

Figure S10. 3D histogram representation of the CS-tensor distribution $\mathrm{P}(\boldsymbol{\delta_{sq}})$, with input parameters $\langle\delta_{11}\rangle = 1650$ ppm, $\langle\delta_{22}\rangle = -4400$ ppm, $\langle\delta_{33}\rangle = -4860$ ppm, $\sigma_{\mathrm{long}} = 2100$ ppm, and $\sigma_{\mathrm{long}} = 600$ ppm, resulting from the evaluation of 10^7 individual CS-tensor $\boldsymbol{\delta_{sq}^{(j)}}$. The considered parameter space was set to include $[-5000,1000]$/ppm for $\delta_{iso}^{(j)}$, [0,18000]/ppm for $\Omega^{(j)}$, and $[-1,1]$ for $\kappa^{(j)}$. Each dimension was equally divided into 100 bins, resulting in 10^6 voxels. A cut-off count of 10 has been included, and the color of each voxel has been rendered to be transparent to ensure comprehensibility.

This is demonstrated in **Figure S10** for the input parameters $\langle\delta_{11}\rangle = 1650$ ppm, $\langle\delta_{22}\rangle = -4440$ ppm, $\langle\delta_{33}\rangle = -4860$ ppm, $\sigma_{\mathrm{long}} = 2100$ ppm, and $\sigma_{\mathrm{eq}} = 600$ ppm. A more convenient graphical representation of the CS-tensor distribution $\mathrm{P}(\boldsymbol{\delta_{sq}})$ is given by the three 2D projections, that are the three bivariate marginal distributions $\mathrm{P}_{\mathrm{m}}^{\kappa}(\delta_{\mathrm{iso}}, \Omega)$, $\mathrm{P}_{\mathrm{m}}^{\delta_{\mathrm{iso}}}(\Omega, \kappa)$, and $\mathrm{P}_{\mathrm{m}}^{\Omega}(\kappa, \delta_{\mathrm{iso}})$ shown in **Figure S11**. Additionally, the respective top panels in **Figure S11** demonstrate the three marginal distributions for the isotropic chemical shift $\mathrm{P}_{\mathrm{m}}(\delta_{\mathrm{iso}})$ in (a), for the span $\mathrm{P}_{\mathrm{m}}(\Omega)$ in (b), and for the skew $\mathrm{P}_{\mathrm{m}}(\kappa)$ in (c). We see that $\mathrm{P}_{\mathrm{m}}(\delta_{\mathrm{iso}})$ and $\mathrm{P}_{\mathrm{m}}(\Omega)$ are indeed Gaussian distributions for the chosen input parameters, while $\mathrm{P}_{\mathrm{m}}(\kappa)$ is asymmetric. We can compute the three average CS-tensor parameters, that are here yielding $\langle\delta_{\mathrm{iso}}\rangle = 2550$ ppm, $\langle\Omega\rangle = 6700$ ppm, and $\langle\kappa\rangle = -0.8$, respectively, indicating the center of $\mathrm{P}(\boldsymbol{\delta_{sq}})$ in **Figure S10**, or equivalently of its marginal distributions in **Figure S11**. Note that while $\langle\delta_{\mathrm{iso}}\rangle$ is indeed given by $\frac{1}{3}(\langle\delta_{11}\rangle + \langle\delta_{22}\rangle + \langle\delta_{33}\rangle)$, $\langle\Omega\rangle$ and $\langle\kappa\rangle$ cannot be directly calculated from $\langle\delta_{11}\rangle$, $\langle\delta_{22}\rangle$, and $\langle\delta_{33}\rangle$, due to the cases where the values of $\Delta_{11}^{(j)}, \Delta_{22}^{(j)}$, and $\Delta_{33}^{(j)}$ need re-ordering.

5.2.2 Correlation in the δ_{iso}-Ω-space. $\langle\delta_{\mathrm{iso}}\rangle$, $\langle\Omega\rangle$ and $\langle\kappa\rangle$ contain information about the average local Pt(II) environment. Information about the Pt(II)-site heterogeneity could now be obtained from the standard deviations for the three average CS-tensor parameters. However, as described in the main text, we found the marginal distribution $\mathrm{P}_{\mathrm{m}}^{\kappa}(\delta_{\mathrm{iso}}, \Omega)$ in the δ_{iso}-Ω-space (**Figure S11**a) to be a more concise description the Pt(II)-site heterogeneity, in part due to the insightful correlation properties of δ_{iso} and Ω.

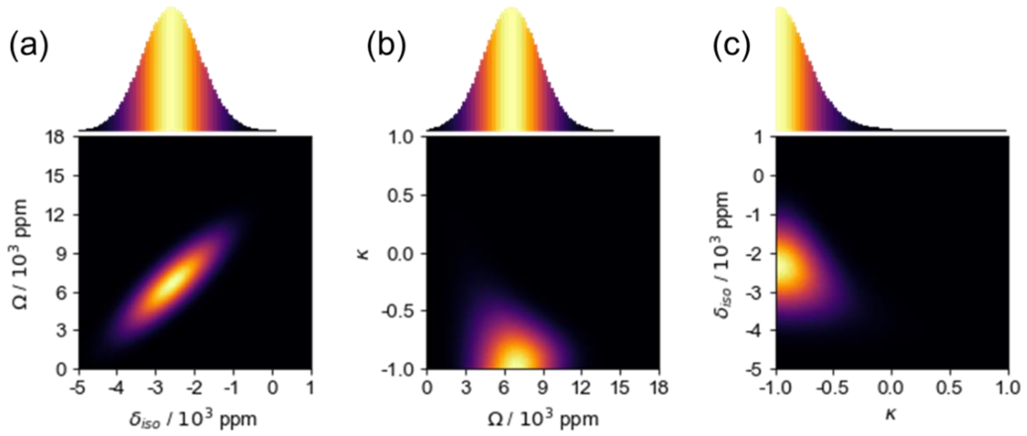

Figure S11. 2D histogram representation of the three marginal distributions (a) $P_m^{\kappa}(\delta_{iso}, \Omega)$, (b) $P_m^{\delta_{iso}}(\Omega, \kappa)$, and (c) $P_m^{\Omega}(\kappa, \delta_{iso})$, computed from the CS-tensor distribution $P(\boldsymbol{\delta_{sq}})$ shown in **Figure S10**. Accordingly, each 2D histogram comprises 10^4 pixels (see caption of **Figure S10**). The top panels show the marginal distributions of the isotropic chemical shift, $P_m(\delta_{iso})$ in (a), for the span $P_m(\Omega)$ in (b), and for the skew $P_m(\kappa)$ in (c).

The correlation coefficient $\rho(\delta_{iso}, \Omega)$ can be calculated from the 10^7 individual CS tensors $\boldsymbol{\delta_{sq}^{(j)}}$ according to

$$\rho(\delta_{iso}, \Omega) = \rho = \frac{\sum_j \left(\delta_{iso}^{(j)} - \langle\delta_{iso}\rangle\right)\left(\Omega^{(j)} - \langle\Omega\rangle\right)}{\sqrt{\sum_j \left(\delta_{iso}^{(j)} - \langle\delta_{iso}\rangle\right)^2 \sum_j \left(\Omega^{(j)} - \langle\Omega\rangle\right)^2}}. \tag{8}$$

We note that if Eq. (7) is a valid approximation, we can likewise approximate the correlation coefficient, which is then given by

$$\rho \approx \sqrt{\frac{\sigma_{long}^2 - \sigma_{eq}^2}{\sigma_{long}^2 + 2\sigma_{eq}^2}}, \tag{9}$$

where we have considered that $\Delta_{11}^{(j)}$, $\Delta_{22}^{(j)}$, and $\Delta_{33}^{(j)}$ are uncorrelated. For a distribution $P(\boldsymbol{\delta_{sq}})$ due to a variation of the $\Delta_{11}^{(j)}$ component only ($\sigma_{long} > \sigma_{eq} = 0$), Eq. (9) is exact, and we observe a perfect correlation $\rho = 1$. Then, all individual CS tensors $\boldsymbol{\delta_{sq}^{(j)}}$ concentrate on a line D with slope $\frac{\Delta\Omega}{\Delta\delta_{iso}} = 3$ in δ_{iso}-Ω-space. This is demonstrated in **Figure S12**a for the same input parameters as described before, but $\sigma_{eq} = 0$. For minor variation of the equatorial components $\Delta_{22}^{(j)}$ and $\Delta_{33}^{(j)}$, such that $\sigma_{long} \gg \sigma_{eq} \neq 0$, as in the example shown in **Figure S10**, the correlation is still significant with $\rho = 0.83$. This is again shown in **Figure S12**b, that corresponds to **Figure S11**a. For the other extreme case, where all components of $\boldsymbol{\delta_{sq}^{(j)}}$ vary equally, $\sigma_{long} = \sigma_{eq}$, no correlation is observed, i.e., $\rho = 0$, as shown in **Figure S12**c. Here all input parameters are again identical to those of **Figure S10**, but $\sigma_{eq} = \sigma_{long} = 2100$ ppm.

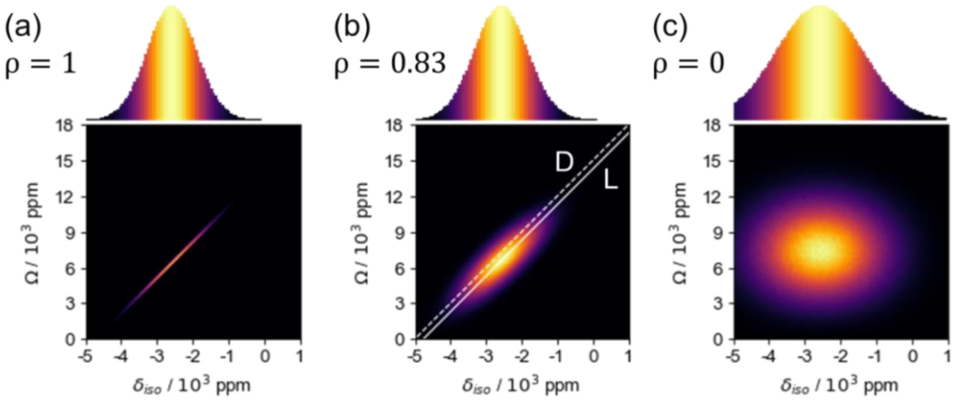

Figure S12. Marginal distributions $P_m^{\kappa}(\delta_{iso}, \Omega)$ and correlation coefficients ρ for input parameters as described for **Figure S10** (see caption), but $\sigma_{eq} = 0$ in (a), $\sigma_{eq} = 600$ ppm (identical to **Figure S10**) in (b), and $\sigma_{long} = \sigma_{eq} = 2100$ ppm in (c). In (b), the characteristic line L (solid), and the $\frac{\Delta\Omega}{\Delta\delta_{iso}} = 3$ line D (dashed) are explicitly indicated (see text). The marginal distributions for $P_m(\delta_{iso})$ are shown in the respective top panels The pixel size is identical to **Figure S11**. The color scale has been adjusted independently for each panel.

<u>5.3.3 The chemical-heterogeneity index σ</u>. Before discussing further the interpretation of ρ, it is important to emphasize that computational and experimental data from this and other studies (see **Section S4**) do indeed confirm the concentration of Pt(II) environments on the line L with slope $\frac{\Delta\Omega}{\Delta\delta_{iso}} = 3$ in δ_{iso}-Ω-space. Further, different Pt(II) sites are located at different positions along L, depending on the nature of the involved ligand atoms. For the analysis of Pt SACs, it is therefore reasonable to define the line L as the dimension of the chemical heterogeneity. The exact location of L in the δ_{iso}-Ω-space is determined by the center of $P_m^{\kappa}(\delta_{iso}, \Omega)$, and thus depends on $\langle\delta_{iso}\rangle$, $\langle\Omega\rangle$, and $\langle\kappa\rangle$. In **Figure S12**b, L is indicated by the solid white line. We can extract the distribution along L, and calculate its standard deviation, that we denote as σ in the main manuscript and use as an index for the chemical heterogeneity. To ensure comparability of σ for distributions with different locations of L, we need to define a common ppm scale. To this end, we introduce a second line D likewise with slope $\frac{\Delta\Omega}{\Delta\delta_{iso}} = 3$. The line D is initially set to run through the origin (0,0) in δ_{iso}-Ω-space, and then shifted in parallel along the direction $\frac{\Delta\Omega}{\Delta\delta_{iso}} = -\frac{1}{3}$, until D traverses either the upper or lower limit for (δ_{iso},Ω) on which $P_m^{\kappa}(\delta_{iso}, \Omega)$ is evaluated. As we have chosen the evaluation interval for Ω ([0,18000]/ppm) to be three times that of δ_{iso} ([-5000,1000]/ppm), D is the diagonal in **Figure S12**b (dashed white line), connecting [-5000,0]/ppm and [1000,18000]/ppm. We define the length of D to reflect the limits for δ_{iso}, i.e., [-5000,1000]/ppm. By projecting L on D, we can calculate σ on a globally defined ppm scale.

<u>5.3.4 The structural-heterogeneity index ρ</u>. For the special case ρ = 1, the marginal distributions $P_m^{\kappa}(\delta_{iso}, \Omega)$ collapse to the characteristic line L (cf. **Figure S12**a and b).

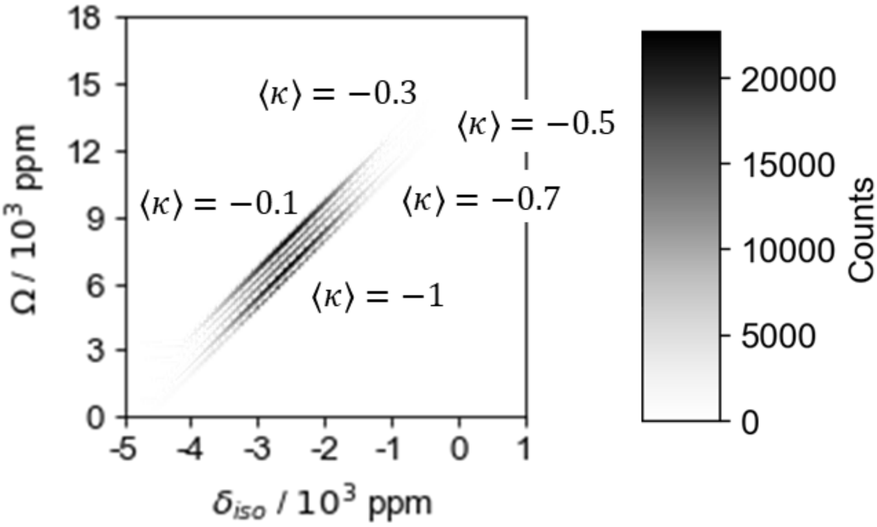

Figure S13. Marginal distributions $P_m^\kappa(\delta_{iso}, \Omega)$ for $\sigma_{long} = 2100$ ppm and $\sigma_{eq} = 0$, $\langle\delta_{11}\rangle = 1650$ ppm, and $\langle\delta_{22}\rangle, \langle\delta_{33}\rangle = -4650, -4650$ ppm ($\langle\kappa\rangle = -1$), $-4230, -5070$ ppm ($\langle\kappa\rangle = -0.7$), $-3810, -5490$ ppm ($\langle\kappa\rangle = -0.5$), $-3390, -5910$ ppm ($\langle\kappa\rangle = -0.3$), and $-2970, -6330$ ppm ($\langle\kappa\rangle = -0.1$). The pixel size is identical to **Figure S11**.

To further understand the interpretation of the correlation coefficient ρ, we have considered a series of marginal distributions $P_m^\kappa(\delta_{iso}, \Omega)$ all with for $\rho = 1$, but with different values for $\langle\kappa\rangle$. Therefore, here we set $\sigma_{long} = 2100$ ppm and $\sigma_{eq} = 0$, and also fixed $\langle\delta_{11}\rangle = 1650$ ppm. It is illustrative to now track the exact position of L for different values of $\langle\delta_{22}\rangle$ and $\langle\delta_{33}\rangle$, and thus different average skew values $\langle\kappa\rangle$. In **Figure S13**, we have shown the $P_m^\kappa(\delta_{iso}, \Omega)$ for $\langle\delta_{22}\rangle, \langle\delta_{33}\rangle = -4650, -4650$ ppm ($\langle\kappa\rangle = -1$), $-4230, -5070$ ppm ($\langle\kappa\rangle = -0.7$), $-3810, -5490$ ppm ($\langle\kappa\rangle = -0.5$), $-3390, -5910$ ppm ($\langle\kappa\rangle = -0.3$), and $-2970, -6330$ ppm ($\langle\kappa\rangle = -0.1$). For clarity, we have changed the color code of the count scale. Following the interpretation detailed above, each of the distributions $P_m^\kappa(\delta_{iso}, \Omega)$ shown in **Figure S13** represent a family of Pt(II) sites with (close-to) identical local geometry and hence $\rho = 1$, but changing chemical environment (chemical heterogeneity only, as observed by the change in $\delta_{iso}^{(j)}$). However, in the presence of several families with different average skew parameters $\langle\kappa\rangle$, we generally observe an expansion along a line perpendicular to L, i.e., a line with a slope of $\frac{\Delta\Omega}{\Delta\delta_{iso}} = -\frac{1}{3}$. Overall, the combination of all $P_m^\kappa(\delta_{iso}, \Omega)$ shown in **Figure S13** is reminiscent of the shape for $P_m^\kappa(\delta_{iso}, \Omega)$ with $\rho < 1$ (cf. **Figure S11**a). We therefore interpret the correlation coefficient as an index for the heterogeneity of the local geometry.

5.3 Comparison with other models

Well-established protocols to model the NMR lineshapes due to a distribution of nuclear sites are the Gaussian isotropic model (GIM, also cf. Czjzek model[27]), and the extended Czjzek model.[22,23] While both models have primarily been applied to describe the NMR lineshapes for quadrupolar nuclei with S > ½, it has been shown that they are likewise applicable to the CS tensor.[22] Following this approach, the CS tensor is given by

$$\boldsymbol{\delta}^{(j)} = \boldsymbol{\delta}^{\mathbf{0}} + \lambda(\epsilon)\widetilde{\boldsymbol{\delta}}^{(j)}, \qquad (10)$$

where λ denotes the scaling factor (called ρ in ref. [22]).The factor λ depends on the input parameter ϵ, which scales the noise contribution $\tilde{\boldsymbol{\delta}}^{(j)}$ according to its norm,

$$\lambda(\epsilon) = \epsilon \frac{\|\boldsymbol{\delta}^{\mathbf{0}}\|}{\|\tilde{\boldsymbol{\delta}}^{(j)}\|}, \tag{11}$$

where $\|\mathbf{A}\| = \sqrt{Tr[\mathbf{A}^2]}$ with Tr denoting the trace. The noise contribution $\tilde{\boldsymbol{\delta}}^{(j)}$ is constructed from six random variables X_k with $\mathrm{k} \in [1,6]$[22], that are assumed to be independent, and identically distributed according to $X_\mathrm{k} \sim \mathcal{N}(0,1)$ leaving four input parameters, namely the parameters $\langle\delta_{11}\rangle$, $\langle\delta_{22}\rangle$, and $\langle\delta_{33}\rangle$ to define $\boldsymbol{\delta}^{\mathbf{0}}$, and ϵ to scale the amount of noise contribution that should be considered. We note that the Czjzek model is closely related to the present model with the confinement that $\sigma_{\mathrm{eq}} = \sigma_{\mathrm{long}}$ for the extended Czjzek model, and further that $\langle\delta_{11}\rangle = \langle\delta_{22}\rangle = \langle\delta_{33}\rangle = 0$ for the original Czjzek model.

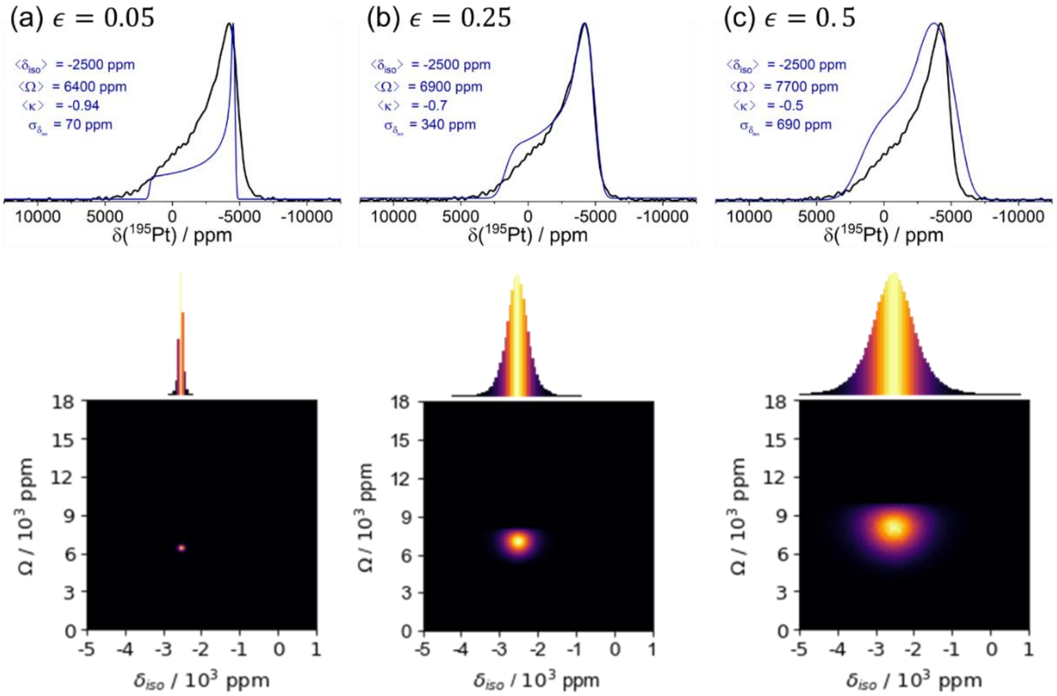

Figure S14. Comparison of the static ^{195}Pt NMR signatures for Pt@NC-15 after the second annealing with lineshapes obtained from the extended Czjzek model. In all cases, $\langle\delta_{11}\rangle = 1700$ ppm, $\langle\delta_{22}\rangle$=$\langle\delta_{33}\rangle = -4600$ ppm, with different amounts of the noise contribution $\epsilon = 0.05$ in (a), $\epsilon = 0.25$ in (b), and $\epsilon = 0.5$ in (c). Bottom panels show the corresponding marginal distributions $\mathrm{P}_\mathrm{m}^{\kappa}(\delta_{\mathrm{iso}}, \Omega)$ and $\mathrm{P}_\mathrm{m}(\delta_{\mathrm{iso}})$. The pixel size is identical to **Figure S11**. The color scale has been adjusted independently for each panel.

In **Figure S14**, we show three examples of ^{195}Pt NMR lineshapes computed according to Eq. (11), and the protocol given in **Section S5.3**. The experimental ^{195}Pt NMR signature corresponds to Pt@NC-15 after the second annealing step. For all examples, we have set $\langle\delta_{11}\rangle = 1700$ ppm, and $\langle\delta_{22}\rangle$=$\langle\delta_{33}\rangle = -4600$ ppm, and varied the varied the amount of the noise contribution $\epsilon = 0.05$ in (a), $\epsilon = 0.25$ in (b), and $\epsilon = 0.5$ in (c). The respective bottom panels show the corresponding marginal distributions $\mathrm{P}_\mathrm{m}^{\kappa}(\delta_{\mathrm{iso}}, \Omega)$ in δ_{iso}-Ω-space, with the marginal distribution $\mathrm{P}_\mathrm{m}(\delta_{\mathrm{iso}})$, i.e., the distribution of isotropic chemical shifts. As output parameters we report the average CS-tensor parameters, as well as the resulting width of $\mathrm{P}_\mathrm{m}(\delta_{\mathrm{iso}})$, $\sigma_{\delta_{\mathrm{iso}}}$. Clearly, none of the produced lineshape can reproduce the experimental signature well.

We note that when modeling the noise contribution with independent, and identically distributed random variables, δ_{iso} and Ω are always uncorrelated (ρ = 0), as likewise displayed by the respective

$P_m^{\kappa}(\delta_{iso}, \Omega)$. With that, **Figure S14** makes a very compelling argument for the lineshape model introduced in **Section S5.2**. From the fast MAS ^{195}Pt NMR spectrum (50 kHz ≈ 600 ppm) shown in **Extended Data Fig. 6**, we know that for the width of the distribution of isotropic chemical shifts, $\sigma_{\delta_{iso}} \gtrsim$ 600 ppm. Without any correlation between δ_{iso} and Ω, as in the (extended) Czjzek model, this produces a broadening incompatible with the relatively narrow low-frequency part of the ^{195}Pt NMR lineshape. This is clearly demonstrated in **Figure S14**c, where $\sigma_{\delta_{iso}}$ is at the order of what would be in agreement with the fast MAS ^{195}Pt NMR spectrum. Conversely, in **Figure S14**b, where the $\sigma_{\delta_{iso}} = 340$ ppm, the broadening fits well with the low-frequency end. However, the distributions of Ω is clearly to narrow to model the continuous lineshape at the high-frequency end, again rationalizing a correlation between the CS-tensor parameters.

5.4 Numerical protocol

In order to compute the NMR signatures associated with CS-tensor distributions $P(\boldsymbol{\delta_{sq}})$, as e.g., shown in **Figure S10**, in principle the individual NMR spectra associated with each voxel ($\boldsymbol{\delta_{sq}}$) can be added up, where each spectrum is weighted according to the relative voxel count. However, in the 3D histogram of $P(\boldsymbol{\delta_{sq}})$ from **Figure S10**, there are 85420 populated voxels, making the computation of the overall NMR signature rather expensive, as this would require computation of 85420 individual powder spectra with different CSA parameters. This brute-force approach would produce a fully converged lineshape for the present model.

To reduce computational costs, we make use of the fact that (1) the NMR lineshape is fully defined by the span and skew (Ω,κ), while δ_{iso} only defines the center of gravity, and (2) we know that a Gaussian distribution of δ_{iso} produces a Gaussian broadening of the NMR line, and as established in Eq. (6) this is indeed the case. Due to (1), all relevant, individual powder patterns associated with a given $P(\boldsymbol{\delta_{sq}})$ are represented by the bivariate marginal distribution $P_m^{\delta_{iso}}(\Omega, \kappa)$ shown in **Figure S11**b, comprising only 4927 populated pixels. For each of these pixels (Ω,κ) we need to determine the center of gravity δ_{iso}, which we can calculate from the position of the line L in the δ_{iso}-Ω-space (**Figure S11**a). Furthermore, we need to apply the correct Gaussian line broadening, which we can also extract from $P_m^{\kappa}(\delta_{iso}, \Omega)$. As described above, in our case for square-planar Pt(II) sites, $P_m^{\kappa}(\delta_{iso}, \Omega)$ is approximately a bivariate Gaussian distribution. The conditional standard deviation σ_{con}, here referring to the standard deviation of δ_{iso} for a given Ω, is constant and defined by

$$\sigma_{con}^2 = (1-\rho)^2 \sigma_{\delta_{iso}}^2, \tag{12}$$

where $\sigma_{\delta_{iso}}$ is the standard deviation for $P_m(\delta_{iso})$, and ρ is the correlation coefficient defined in Eq. (8). The full routine can be summarized as follows:

I. Choice of input parameters $\langle\delta_{11}\rangle$, $\langle\delta_{22}\rangle$, $\langle\delta_{33}\rangle$, σ_{long}, and σ_{eq}
II. Calculating marginal distributions $P_m^{\kappa}(\delta_{iso}, \Omega)$, $P_m^{\delta_{iso}}(\Omega, \kappa)$, and $P_m(\delta_{iso})$, average CS-tensor parameters $\langle\delta_{iso}\rangle$, $\langle\Omega\rangle$, and $\langle\kappa\rangle$, as well as Q_{ICE} parameters σ and ρ
III. Computation of the overall lineshape:
    a. The ^{195}Pt time-domain NMR signal associated with the CSA powder pattern for each populated pixel (Ω,κ) with $\delta_{iso} = 0$ ppm is computed.
    b. Each signal is scaled due to the respective relative pixel count, and multiplied with a Gaussian function to introduce a Gaussian line broadening in the frequency domain according to Eq. (12).
    c. All signals are Fourier transformed, shifted towards the correct center of gravity δ_{iso} based on the position of the line L, and added together.

We note that some of these steps can be performed in either the time or frequency domain. Furthermore, for the extreme case $\rho = 0$, as e.g., shown in **Figure S12**c, the individual frequency-domain spectra do not need to be shifted according to the line L, but can all independently shifted towards $\langle\delta_{\text{iso}}\rangle$. Also, then $\sigma_{\text{con}} = \sigma_{\delta_{\text{iso}}}$ (see Eq. (12)). To further accelerate the computation, step IIIa has been accelerated either (i) by pre-calculating a library of ^{195}Pt time-domain NMR signals for 10^5 combination combinations (Ω,κ) using the SIMPSON simulation software[28], or (ii) by parallelizing the calculation of different crystallites in using GPU acceleration. In both cases, triangular interpolation is used.[29]
Using this optimized approach provides the same fully converged lineshape as the brute-force method outlined above, but with a 10-20 fold time-saving due to the smart handling of the isotropic shift distribution. With the present implementation in Python, calculation of a converged lineshape lasts 1-2 min on a standard office computer.

However, an actual optimization rather than a modeling of the experimental lineshapes by hand, let alone the calculation of confidence intervals for the input parameters (see **Section S5.5**), typically requires hundreds to thousands of iterations, in which case minute-lasting calculations are prohibitively long. In order to further accelerate the simulations, we have therefore carefully evaluated the convergence of the numerically produced lineshapes with respect to the number of included individual powder spectra. To this end, we first chose a series of different input-parameter sets $\langle\delta_{11}\rangle, \langle\delta_{11}\rangle, \langle\delta_{11}\rangle, \sigma_{\text{eq}}$, and σ_{long}, covering the expected ranges of parameters for the studied SACs. The corresponding fully-converged lineshapes were used as computational references, for which the exact set of input parameters are known. We then reduced the number of individual powder spectra used for the lineshape calculation and found that as few as 1000 individual powder patterns are sufficient to reproduce the computational references. We note that we always checked the agreement between the spectra resulting from the reduced distribution and the fully converged approach. By implementing this approach in EasyNMR[30] (https://easy.csdm.dk), and using GPU-accelerated calculations of the lineshapes, one simulation could be done in 900 ms on a regular laptop, demonstrating further reduction of computational costs by roughly two orders of magnitude compared to the algorithm providing the fully converged lineshape. We emphasize that all optimizations required to compute the parameters listed in **Table S13**Table **S14**, and the confidence intervals discussed in **Section S5.5** have been achieved using ca 1000 optimizations corresponding to ca 300 000 lineshape simulations (and thus 300 million CSA powder patterns). Using the fast approach, this has lasted roughly 75 hours on a laptop, whereas the same would have lasted 7500 hours (close to 1 year) using the fully converged lineshapes.

5.5 Uniqueness and stability

With the availability of the very fast lineshape simulations described in the previous section, we are now able to further validate the numerical model proposed herein. To demonstrate the uniqueness of the reported sets of parameters, i.e., that they correspond to a global minimum in the five-dimensional parameter space, we have extended our numerical analysis to compute the 95 % confidence intervals for each of the input parameters $\langle\delta_{11}\rangle$, $\langle\delta_{22}\rangle$,$\langle\delta_{33}\rangle$, σ_{long}, and σ_{eq}. To do this, we first determined the optimum set of parameters $x_0 = \left(\langle\delta_{11}\rangle, \langle\delta_{22}\rangle, \langle\delta_{33}\rangle, \sigma_{\text{eq}}, \sigma_{\text{long}}\right)_{\text{opt}}$ associated with the minimum value $\chi_0^2 = \chi^2(x_0)$. Then, one of the five input parameters was set to a fixed value, while the remaining four parameters were optimized to obtain the best fit (this parameter set is just labelled x), providing a minimum value of $\chi^2(x)$. The optimization is then repeated for various fixed values, covering a reasonable range of inputs. This is demonstrated in **Figure S15**, which shows the repeated optimization for different fixed values of (a) $\langle\delta_{11}\rangle$, (b) $\langle\delta_{22}\rangle$, (c) σ_{eq}, and (d) σ_{long} against the ^{195}Pt NMR spectrum of Pt@NC-15 after the second annealing step. Each of the black circles indicate the minimum value of χ^2 obtained after full optimization, where the global optimum parameter set x_0 is indicated in green. Clearly, in **Figure S15**a, c, and d, the circles describe a parabolic behaviour, that can be fitted using

$$\chi^2_{est}(x) = a(x - x_0)^2 + \chi^2_0, \tag{13}$$

shown as a blue line in **Figure S15**. In Eq. (13), a denotes the curvature, which can be used to calculate the 95 % confidence interval for the respective input parameter according to $C_{95\%} = 2/\sqrt{a}$.[31] All optimum parameters and 95 % confidence intervals are given in **Figure S15**. The confidence intervals are again summarized in **Table S13**.

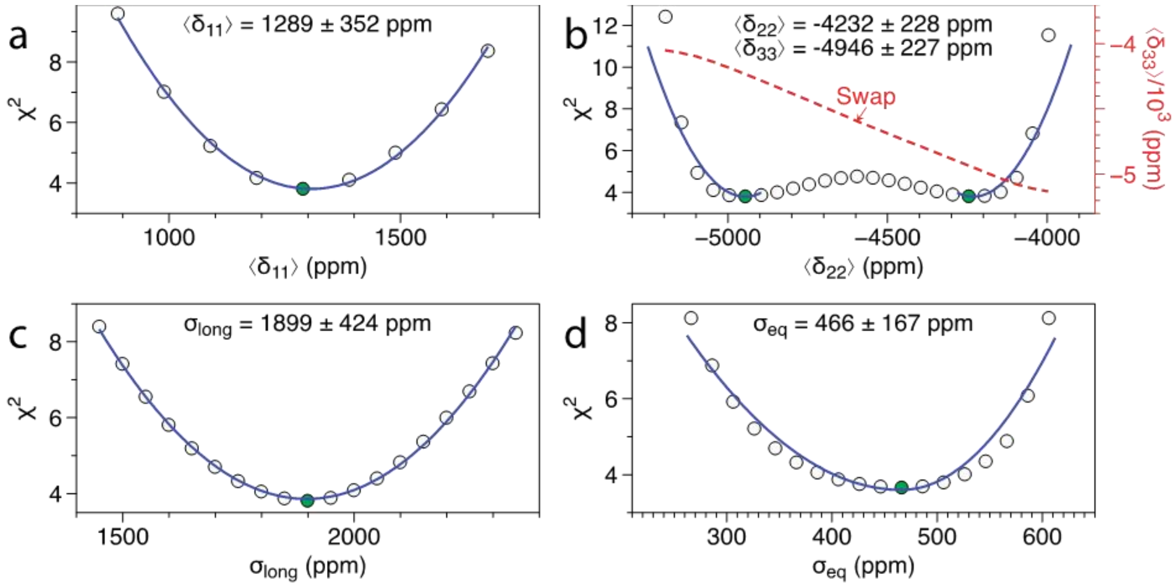

Figure S15. 95 % confidence interval calculations. χ^2 values are reported for the fits of the ^{195}Pt NMR spectrum of Pt@NC-15 after the second annealing step. The spectrum was fitted based on the input parameters $\langle\delta_{11}\rangle$, $\langle\delta_{22}\rangle$,$\langle\delta_{33}\rangle$, σ_{long}, and σ_{eq}. Each data point (black circles) represents a fitting where the value indicated at the x axis is fixed at the specific value, but with the other parameters optimized. For all graphs, the black circles show the result of the fit for the given value of (a) $\langle\delta_{11}\rangle$, (b) $\langle\delta_{22}\rangle$, (c) σ_{long}, and (d) σ_{eq}. The solid blue lines represent the fit parabolic curve given in Eq. (13). The global optimum parameter set is labelled in green. In (b), the two parameters $\langle\delta_{22}\rangle$ and $\langle\delta_{33}\rangle$ are swapped at a value of around -4595 ppm as indicated by the arrow. For illustration, $\langle\delta_{33}\rangle$ resulting from the fitting is shown in red on the right y axis.

The analysis for the input parameter $\langle\delta_{22}\rangle$, demonstrated in **Figure S15**b, has a particular feature and requires special considerations. The optimum parameter set x_0 involves the first minimum observed at $\langle\delta_{22}\rangle$ = - 4232 ppm. Further increasing $\langle\delta_{22}\rangle$ from -4232 ppm towards 0 ppm shows a clear sharp edge as expected. Moreover, while a decrease of $\langle\delta_{22}\rangle$ generally results in higher values of χ^2, a second minimum is found at $\langle\delta_{22}\rangle$ = -4946 ppm. Upon forcing a decrease of $\langle\delta_{22}\rangle$, and optimizing $\langle\delta_{11}\rangle$, $\langle\delta_{33}\rangle$, σ_{long}, and σ_{eq}, the components $\langle\delta_{22}\rangle$ and $\langle\delta_{33}\rangle$ are swapped by the algorithm. To demonstrate this, the optimum value for the $\langle\delta_{33}\rangle$ component is shown in red in **Figure S15**b. This parameter varies linearly between the two minima. The local maximum at $\langle\delta_{22}\rangle$ = -4595 ppm indicates the swap of the two components, and is indicated with the arrow. It is important to note that all parameter sets x enclosed by the two minima come in identical pairs, symmetrically flanking the local maximum at $\langle\delta_{22}\rangle$ = -4595 ppm. These pairs describe the same average CS tensor, and of course result in identical NMR lineshapes. Therefore, the optimum parameter sets for $\langle\delta_{22}\rangle$=-4232 ppm and $\langle\delta_{22}\rangle$=-4946 ppm are likewise identical, and both corresponds to x_0. We further point out, that **Figure S15**b generally reflects the high symmetry of the square-planar coordination for the local Pt(II) sites. While for the local maximum at $\langle\delta_{22}\rangle$ = -4595 ppm, $\langle\delta_{22}\rangle = \langle\delta_{33}\rangle$, and thus $\langle\kappa\rangle = -1$, the optimum parameter set x_0, where $\langle\kappa\rangle = -0.7$, corresponds to minor symmetry deviations on average. Any further deviations result in very high values of χ^2, as demonstrated by the sharp parabolic edges above $\langle\delta_{22}\rangle$=-4232 ppm, and below $\langle\delta_{22}\rangle$=-4946 ppm. The best parabolic fit found according to Eq. (13) shows a compromise

between these clear edges, and less sharp edge towards the local maximum at $\langle\delta_{22}\rangle$ = -4595 ppm.

Overall, **Figure S15** clearly demonstrates the existence of a global minimum in the five-dimensional input-parameter space, that corresponds to a unique set of input parameters, and thus a unique numerical lineshape.

Based on the 95 % confidence intervals for the input parameters $\langle\delta_{11}\rangle$, $\langle\delta_{22}\rangle$, $\langle\delta_{33}\rangle$, σ_{long}, and σ_{eq} shown in **Figure S15**, we can now in principle likewise calculate the 95 % confidence intervals for the output parameters $\langle\delta_{\mathrm{iso}}\rangle$, $\langle\Omega\rangle$, $\langle\kappa\rangle$, σ, and ρ. Assuming that there exists a function f_u, relating input parameters *k* and output parameters *u*, the 95 % confidence interval for an output parameter *u* can be calculated according to

$$C_{95\%}[f_u] = \sqrt{\sum_k \left(\frac{\partial f_u}{\partial x_k}\right)^2 C^2_{95\%}(x_k)}\,. \tag{14}$$

For the average CS-tensor parameters $\langle\delta_{\mathrm{iso}}\rangle$, $\langle\Omega\rangle$, and $\langle\kappa\rangle$, the respective functions f_u are given in Eq. (5). We note that for $\langle\delta_{\mathrm{iso}}\rangle$, the function $f_{\langle\delta_{\mathrm{iso}}\rangle}$ according to Eq. (5) is exact, while Eq. (5) is a very close approximation for $\langle\Omega\rangle$ and $\langle\kappa\rangle$ due to potential re-ordering of the components $\Delta'^{(j)}_{11}$, $\Delta'^{(j)}_{22}$, and $\Delta'^{(j)}_{33}$. Furthermore, there are no exact analytical functions f_u for σ and ρ. Yet, again, we can find very close approximations of f_σ by using the standard deviation of $\langle\delta_{\mathrm{iso}}\rangle$ given in Eq. (6), and of f_ρ by using Eq. (9). The 95 % confidence intervals for all input and output parameters are summarized in **Table S13**.

Table S13. Summary of the 95 % confidence intervals and reported precision for input and output parameters.

	Parameter	95 % confidence interval	Reported precision
Input	$\langle\delta_{11}\rangle$	± 352 ppm	-
	$\langle\delta_{22}\rangle$	± 228 ppm	-
	$\langle\delta_{33}\rangle$	± 227 ppm	-
	σ_{long}	± 424 ppm	-
	σ_{eq}	± 167 ppm	-
Output	$\langle\delta_{\mathrm{iso}}\rangle$	± 159 ppm	100 ppm
	$\langle\Omega\rangle$	± 419 ppm	500 ppm
	$\langle\kappa\rangle$	± 0.14	0.1
	σ	± 136 ppm	100 ppm
	ρ	± 0.07	0.1

While the 95 % confidence intervals do not directly represent the precision of the individual parameters, they merely represent statistical estimates of the likelihood of a given parameter. However, we find that the 95 % confidence intervals roughly match the precision of the parameters as based on the hundreds of optimizations and visual agreement between simulations and experiments. Hence, we use the 95 % confidence intervals to derive a reasonable precision for the parameters shown in the main text, as listed in **Table S13**. All exact and rounded parameters are summarized in **Table S13**.

We emphasize that the changes of parameters that we observe for different synthetic protocols, for different supports, and during the reaction remain notably larger than the reported precision, and thereby are clearly significant.

5.6 Summary of parameters used for the studied Pt SAC samples

Table S14: Summary of the input and output parameters corresponding to the lineshapes reported in the main text.

Sample	Pt@NC-5		Pt@NC-15		Pt@NC-15	Pt@PTI	Pt@NC-1			$Pt@SiO_2$
Annealing step	1st ann	2nd ann	1st ann	2nd ann	2nd ann	2nd ann	pristine[&]	1 h[&]	12 h[&]	-
Input	Exact values									
$\langle\delta_{11}\rangle$ / ppm	4143	2310	2452	1289	1589	1890	2454	3200	4417	880
$\langle\delta_{22}\rangle$ / ppm	-4335	-4053	-4344	-4232	-4151	3617	-4074	-3945	-4031	-4779
$\langle\delta_{33}\rangle$ / ppm	-5131	-5044	-5016	-4946	-5005	5890	-5049	-5205	-5088	-7800
σ_{long} / ppm	1770	1073	1153	1899	2062	1745	1869	1903	1666	1981
σ_{eq} / ppm	587	518	471	466	398	1905	544	477	687	1023
Output	Exact values									
$\langle\delta_{iso}\rangle$ /ppm	-1774	-2262	-2309	-2630	-2517	-2535	-2081	-1983	-1567	-3900
$\langle\Omega\rangle$ / ppm	9379	7384	7511	6282	6660	8027	7875	8403	9560	8686
$\langle\kappa\rangle$	-0.78	-0.72	-0.79	-0.72	-0.70	-0.31	-0.70	-0.69	-0.75	-0.28
σ / ppm	620	375	395	652	707	683	661	670	593	845
ρ	0.75	0.58	0.72	0.87	0.89	0.08	0.81	0.86	0.67	0.53
Output	Within reported precision									
$\langle\delta_{iso}\rangle$ / ppm	-1800	-2300	-2300	-2600	-2500	-2500	-2100	-2000	-1600	-3900
$\langle\Omega\rangle$ / ppm	-9500	7500	7500	6500	6500	8000	8000	8500	9500	8500
$\langle\kappa\rangle$	-0.8	-0.7	-0.8	-0.7	-0.7	-0.3	-0.7	-0.7	-0.8	-0.3
σ / ppm	600	400	400	700	700	700	700	700	600	800
ρ	0.8	0.6	0.7	0.9	0.9	0.1	0.8	0.9	0.7	0.5

[&]After the second annealing step. Pristine, 1h, and 12h here refer to the time on stream.

6. References

(1) Artyushkova, K. Misconceptions in Interpretation of Nitrogen Chemistry from X-Ray Photoelectron Spectra. *J. Vac. Sci. Technol. A* **2020**, *38* (3), 031002. https://doi.org/10.1116/1.5135923.

(2) Lin, R.; Kaiser, S. K.; Hauert, R.; Pérez-Ramírez, J. Descriptors for High-Performance Nitrogen-Doped Carbon Catalysts in Acetylene Hydrochlorination. *ACS Catal.* **2018**, *8* (2), 1114–1121. https://doi.org/10.1021/acscatal.7b03031.

(3) Naumkin, A. V.; Kraut-Vass, A.; Gaarenstroom, S. W.; Powell, C. J. NIST X-Ray Photoelectron Spectroscopy Database, NIST Standard Reference Database Number 20. *National Institute of Standards and Technology*; Gaithersburg, 2000.

(4) Ravel, B.; Newville, M. ATHENA, ARTEMIS, HEPHAESTUS: Data Analysis for X-Ray Absorption Spectroscopy Using IFEFFIT. *J. Synchrotron Radiat.* **2005**, *12* (4), 537–541. https://doi.org/10.1107/S0909049505012719.

(5) O'Dell, L. A.; Schurko, R. W. QCPMG Using Adiabatic Pulses for Faster Acquisition of Ultra-Wideline NMR Spectra. *Chem. Phys. Lett.* **2008**, *464* (1–3), 97–102. https://doi.org/10.1016/j.cplett.2008.08.095.

(6) Koppe, J.; Bußkamp, M.; Hansen, M. R. Frequency-Swept Ultra-Wideline Magic-Angle Spinning NMR Spectroscopy. *J. Phys. Chem. A* **2021**, *125* (25), 5643–5649. https://doi.org/10.1021/acs.jpca.1c02958.

(7) Hung, I.; Gan, Z. On the Practical Aspects of Recording Wideline QCPMG NMR Spectra. *J. Magn. Reson.* **2010**, *204* (2), 256–265. https://doi.org/10.1016/j.jmr.2010.03.001.

(8) Harris, F. J. On the Use of Windows for Harmonic Analysis with the Discrete Fourier Transform. *Proc. IEEE* **1978**, *66* (1), 51–83. https://doi.org/10.1109/PROC.1978.10837.

(9) Slichter, C. P. NMR Study of Platinum Catalysts. *Surf. Sci.* **1981**, *106* (1), 382–396. https://doi.org/10.1016/0039-6028(81)90226-0.

(10) Rhodes, H. E.; Wang, P.-K.; Makowka, C. D.; Rudaz, S. L.; Stokes, H. T.; Slichter, C. P.; Sinfelt, J. H. NMR of Platinum Catalysts. II. Relaxation. *Phys. Rev. B* **1982**, *26* (7), 3569–3574. https://doi.org/10.1103/PhysRevB.26.3569.

(11) Rhodes, H. E.; Wang, P.-K.; Stokes, H. T.; Slichter, C. P.; Sinfelt, J. H. NMR of Platinum Catalysts. I. Line Shapes. *Phys. Rev. B* **1982**, *26* (7), 3559–3568. https://doi.org/10.1103/PhysRevB.26.3559.

(12) Ansermet, J.-Ph.; Slichter, C. P.; Sinfelt, J. H. Solid State NMR Techniques for the Study of Surface Phenomena. *Prog. Nucl. Magn. Reson. Spectrosc.* **1990**, *22* (5), 401–421. https://doi.org/10.1016/0079-6565(90)80005-3.

(13) Townes, C. H.; Herring, C.; Knight, W. D. The Effect of Electronic Paramagnetism on Nuclear Magnetic Resonance Frequencies in Metals. *Phys. Rev.* **1950**, *77* (6), 852–853. https://doi.org/10.1103/PhysRev.77.852.

(14) Korringa, J. Nuclear Magnetic Relaxation and Resonnance Line Shift in Metals. *Physica* **1950**, *16* (7), 601–610. https://doi.org/10.1016/0031-8914(50)90105-4.

(15) te Velde, G.; Bickelhaupt, F. M.; Baerends, E. J.; Fonseca Guerra, C.; van Gisbergen, S. J. A.; Snijders, J. G.; Ziegler, T. Chemistry with ADF. *J. Comput. Chem.* **2001**, *22* (9), 931–967. https://doi.org/10.1002/jcc.1056.

(16) Autschbach, J. The Role of the Exchange-Correlation Response Kernel and Scaling Corrections in Relativistic Density Functional Nuclear Magnetic Shielding Calculations with the Zeroth-Order Regular Approximation. *Mol. Phys.* **2013**, *111* (16–17), 2544–2554. https://doi.org/10.1080/00268976.2013.796415.

(17) van Lenthe, E.; Snijders, J. G.; Baerends, E. J. The Zero-order Regular Approximation for Relativistic Effects: The Effect of Spin–Orbit Coupling in Closed Shell Molecules. *J. Chem. Phys.* **1996**, *105* (15), 6505–6516. https://doi.org/10.1063/1.472460.

(18) van Lenthe, E.; van Leeuwen, R.; Baerends, E. J.; Snijders, J. G. Relativistic regular two-component Hamiltonians. *Int. J. Quantum Chem.* **1996**, *57* (3), 281–293. https://doi.org/10.1002/(SICI)1097-461X(1996)57:3<281::AID-QUA2>3.0.CO;2-U.

(19) Venkatesh, A.; Gioffrè, D.; Atterberry, B. A.; Rochlitz, L.; Carnahan, S. L.; Wang, Z.; Menzildjian, G.; Lesage, A.; Copéret, C.; Rossini, A. J. Molecular and Electronic Structure of Isolated Platinum Sites Enabled by the Expedient Measurement of 195Pt Chemical Shift Anisotropy. *J. Am. Chem. Soc.* **2022**, *144* (30), 13511–13525. https://doi.org/10.1021/jacs.2c02300.
(20) Wang, Z.; Völker, L. A.; Robinson, T. C.; Kaeffer, N.; Menzildjian, G.; Jabbour, R.; Venkatesh, A.; Gajan, D.; Rossini, A. J.; Copéret, C.; Lesage, A. Speciation and Structures in Pt Surface Sites Stabilized by N-Heterocyclic Carbene Ligands Revealed by Dynamic Nuclear Polarization Enhanced Indirectly Detected 195Pt NMR Spectroscopic Signatures and Fingerprint Analysis. *J. Am. Chem. Soc.* **2022**, *144* (47), 21530–21543. https://doi.org/10.1021/jacs.2c08300.
(21) Lucier, B. E. G.; Reidel, A. R.; Schurko, R. W. Multinuclear Solid-State NMR of Square-Planar Platinum Complexes — Cisplatin and Related Systems. *Can. J. Chem.* **2011**, *89* (7), 919–937. https://doi.org/10.1139/v11-033.
(22) Vasconcelos, F.; Cristol, S.; Paul, J.-F.; Delevoye, L.; Mauri, F.; Charpentier, T.; Caër, G. L. Extended Czjzek Model Applied to NMR Parameter Distributions in Sodium Metaphosphate Glass. *J. Phys. Condens. Matter* **2013**, *25* (25), 255402. https://doi.org/10.1088/0953-8984/25/25/255402.
(23) Caër, G. L.; Bureau, B.; Massiot, D. An Extension of the Czjzek Model for the Distributions of Electric Field Gradients in Disordered Solids and an Application to NMR Spectra of 71Ga in Chalcogenide Glasses. *J. Phys. Condens. Matter* **2010**, *22* (6), 065402. https://doi.org/10.1088/0953-8984/22/6/065402.
(24) de Oliveira, M. Jr.; Aitken, B.; Eckert, H. Structure of P2O5–SiO2 Pure Network Former Glasses Studied by Solid State NMR Spectroscopy. *J. Phys. Chem. C* **2018**, *122* (34), 19807–19815. https://doi.org/10.1021/acs.jpcc.8b06055.
(25) Shaltiel, D.; Low, W. Anisotropic Broadening of Linewidth in the Paramagnetic Resonance Spectra of Magnetically Dilute Crystals. *Phys. Rev.* **1961**, *124* (4), 1062–1067. https://doi.org/10.1103/PhysRev.124.1062.
(26) Herzfeld, J.; Berger, A. E. Sideband Intensities in NMR Spectra of Samples Spinning at the Magic Angle. *J. Chem. Phys.* **1980**, *73* (12), 6021–6030. https://doi.org/10.1063/1.440136.
(27) Czjzek, G.; Fink, J.; Götz, F.; Schmidt, H.; Coey, J. M. D.; Rebouillat, J.-P.; Liénard, A. Atomic Coordination and the Distribution of Electric Field Gradients in Amorphous Solids. *Phys. Rev. B* **1981**, *23* (6), 2513–2530. https://doi.org/10.1103/PhysRevB.23.2513.
(28) Bak, M.; Rasmussen, J. T.; Nielsen, N. C. SIMPSON: A General Simulation Program for Solid-State NMR Spectroscopy. *J. Magn. Reson.* **2000**, *147* (2), 296–330. https://doi.org/10.1006/jmre.2000.2179.
(29) Alderman, D. W.; Solum, M. S.; Grant, D. M. Methods for Analyzing Spectroscopic Line Shapes. NMR Solid Powder Patterns. *J. Chem. Phys.* **1986**, *84* (7), 3717–3725. https://doi.org/10.1063/1.450211.
(30) Juhl, D. W.; Tošner, Z.; Vosegaard, T. Chapter One - Versatile NMR Simulations Using SIMPSON. In *Annual Reports on NMR Spectroscopy*; Webb, G. A., Ed.; Academic Press, 2020; Vol. 100, pp 1–59. https://doi.org/10.1016/bs.arnmr.2019.12.001.
(31) Vosegaard, T.; Hald, E.; Langer, V.; Skov, H. J.; Daugaard, P.; Bildsøe, H.; Jakobsen, H. J. Improved Hardware and Software for Single-Crystal NMR Spectroscopy. *J. Magn. Reson.* **1998**, *135* (1), 126–132. https://doi.org/10.1006/jmre.1998.1551.
